# Mapping Normative Muscle Health Metrics Across the Aging Continuum: A Multinational Study Pooling Data From Eight Cohorts in Japan, Malaysia and Taiwan

**DOI:** 10.1002/jcsm.13731

**Published:** 2025-02-19

**Authors:** Liang‐Kung Chen, Lin‐Chieh Meng, Li‐Ning Peng, Wei‐Ju Lee, Shu Zhang, Yukiko Nishita, Rei Otsuka, Minoru Yamada, Wen‐Harn Pan, Shahrul Bahyah Kamaruzzaman, Jean Woo, Fei‐Yuan Hsiao, Hidenori Arai

**Affiliations:** ^1^ Center for Geriatrics and Gerontology Taipei Veterans General Hospital Taipei Taiwan; ^2^ Center for Healthy Longevity and Aging Sciences National Yang Ming Chiao Tung University Taipei Taiwan; ^3^ Taipei Municipal Gan‐Dau Hospital (Managed by Taipei Veterans General Hospital) Taipei Taiwan; ^4^ Graduate Institute of Clinical Pharmacy, College of Medicine National Taiwan University Taipei Taiwan; ^5^ Department of Family Medicine Taipei Veterans General Hospital Yuanshan Branch Yi‐Lan Taiwan; ^6^ Department of Epidemiology of Aging, Center for Gerontology and Social Science National Center for Geriatrics and Gerontology Obu Japan; ^7^ Faculty of Human Sciences University of Tsukuba Tokyo Japan; ^8^ Institute of Biomedical Sciences Academia Sinica Taipei Taiwan; ^9^ Department of Medicine, Faculty of Medicine University of Malaya Kuala Lumpur Malaysia; ^10^ Department of Medicine and Therapeutics, Faculty of Medicine The Chinese University of Hong Kong Hong Kong SAR China; ^11^ School of Pharmacy, College of Medicine National Taiwan University Taipei Taiwan; ^12^ Department of Pharmacy National Taiwan University Hospital Taipei Taiwan; ^13^ Department of Epidemiology of Aging, Research Institute National Center for Geriatrics and Gerontology Obu Aichi Japan

**Keywords:** aging, Asian population, muscle health metrics, physical performance decline, sex‐specific reference centiles

## Abstract

**Background:**

The vigour of our musculature wanes as the years advance, and prognosticating the concomitant trajectories throughout the course of life assumes paramount importance for judicious and timely interventions. In the present study, we aimed to establish age‐ and sex‐specific reference centiles for multiple muscle health metrics and reveal the distributions of these metrics throughout the aging process in the Asian population.

**Methods:**

By using cross‐sectional pooled data of community dwellers aged 20 years or older in eight cohorts from Taiwan, Japan and Malaysia, normative values for muscle health metrics (calf circumference (cm), relative appendicular skeletal muscle (RASM) (kilogram per square metre), body mass index (BMI)–adjusted appendicular skeletal muscle mass (kilogram/(kilogram per square metre)), handgrip strength (kilogram), five‐time chair stand (seconds) and gait speed (metre per second)) in men and women, categorized by age groups, are calculated. The mean values, along with the 5th, 25th, 50th, 75th and 95th percentiles of these muscle health metrics, are also delineated for both sexes.

**Results:**

Among 34 265 (16 164 men, 18 101 women) participants from eight cohorts, calf circumference declined in age groups from 60 years onward. RASM values declined from the 50s in men but were stable in women until the 80s. ASM/BMI values showed declines in older age groups for both sexes. Handgrip strength declined similarly from 40 years of age in both sexes. Five‐time chair stand performance declined from the 30s. Gait speed peaked at 1.6 m/s in men in their 50s and then declined, while it declined in women in their 60s. The inflection points for decline differed by metric and sex. The 20th percentile cutoffs for individuals aged 65–69 years were as follows: calf circumference, 33.0 cm (men) and 31.5 cm (women); RASM, 7.0 kg/m^2^ (men) and 5.5 kg/m^2^ (women); ASM/BMI, 0.78 kg/(kg/m^2^) (men) and 0.56 kg/(kg/m^2^) (women); handgrip strength, 30.4 kg (men) and 18.1 kg (women); five‐time chair stand, 9.4 s (men) and 10.0 s (women); and gait speed, 0.9 m/s (both). Those in the fifth percentile of all muscle health metrics faced earlier declines than their 95th percentile counterparts did, highlighting the critical roles in identifying these high‐risk groups.

**Conclusion:**

The pooled analysis of eight Asian cohorts clearly outlined the age‐related changes in various muscle health metrics, with the inflection point of accelerated decline showing age‐ and sex‐specific characteristics. Defining trajectories of muscle health metrics across life stages facilitates timely interventions to mitigate age‐related risks and promote healthy longevity.

## 1 Introduction

Aging is a complex process characterized by dysregulated homeostasis, diminished physiological reserve, declining organ functions, impaired physical and cognitive abilities, increased multimorbidity and heightened social vulnerability. These factors intertwine over time, leading to adverse impacts later in life [1, 2, 3, 4]. Changes in body composition, a prominent aspect of aging, have significant implications for overall health and functional capacity [5, 6]. Aging typically leads to a decrease in muscle and bone mass and an increase in body fat, which can exacerbate chronic conditions such as diabetes, cardiovascular disease, osteoporosis and sarcopenia [5, 6, 7]. Changes in body composition not only affect physical appearance but also contribute to a plethora of health issues, including reduced mobility, increased risk of falls and fractures, metabolic disturbances, compromised immune function, disability, dementia and mortality later in life [7, 8].

Specifically, sarcopenia, typified by an age‐related decrease in muscle mass concomitant with reduced muscle function [9, 10], has been shown to correlate with various adverse outcomes in older individuals [11, 12]. The advent of the ICD‐10‐CM code for sarcopenia, coupled with the development of various pharmaceutical agents, has substantially enhanced the global recognition of sarcopenia [13]. However, the existing limitations in reversing sarcopenia through pharmaceutical interventions pose a considerable management dilemma [14, 15]. To date, the most efficacious strategy for sarcopenia management is the combination of nutritional supplementation and physical exercise [16, 17]. Gradually, skeletal muscle health has taken an increasingly central stage in recent research because of its extensive interplay with various organs and its potential to alter the trajectory of healthy aging. Skeletal muscle, a key endocrine organ that secretes myokines, interacts with various organs, influencing aging and mitigating conditions such as osteoporosis, diabetes, atherosclerosis and cognitive decline [18, 19].

In addition to the diagnosis and treatment of sarcopenia, the promotion of muscle health, which involves elements such as mass, composition, strength, performance and metabolism, is highly important for fostering healthy longevity [10, 20]. Therefore, adopting an approach for muscle health metrics from an early age could bolster physiological reserves and mitigate age‐related health risks. In addition, leveraging normative muscle health metrics from multiple international cohorts, researchers and clinicians can proactively initiate early lifestyle modifications, adjust therapeutic choices and set goals for chronic conditions, thereby pursuing optimal late‐life wellbeing earlier in the life course. It is crucial to acknowledge the ethnic‐specific nuances in these metrics, given the variations in body composition observed across diverse ethnic populations. Nevertheless, existing studies addressing this issue are lacking. Only very few studies have been conducted in a single Asian country or focused on a single metric of muscle health (mainly handgrip strength) rather than comprehensively evaluating all relevant muscle health metrics [21, 22].

Therefore, we aim to establish age‐ and sex‐specific reference centiles for multiple muscle health metrics (calf circumference), relative appendicular skeletal muscle (RASM), body mass index–adjusted appendicular skeletal muscle mass (ASM/BMI), handgrip strength, five‐time chair stand and gait speed and reveal the distribution of these metrics throughout the aging process via pooled data from community dwellers aged 20 years or older in eight cohorts from Japan, Malaysia and Taiwan.

## 2 Methods

### 2.1 Data Source

This cross‐sectional study collected data from eight cohorts in Taiwan, Japan and Malaysia, with a primary focus on middle‐aged and older individuals and additional data from adolescents and younger adults. These include two prospective longitudinal cohort studies in Taiwan, the I‐Lan Longitudinal Aging Study (ILAS) [23] and the Longitudinal Aging Study of Taipei (LAST) [24]; two nationally representative surveys including the Taiwan Longitudinal Study of Aging (TLSA) [25] and the Nutrition and Health Survey in Taiwan (NAHSIT) [26]; and one implementation research study, the Gan‐Dau Healthy Longevity Plan [27]. Additionally, two prospective longitudinal cohort studies were included from Japan, the mixed cohort from the MUSCLE study and the Tango study [28], and the National Institute for Longevity Sciences Longitudinal Study of Aging (NILS‐LSA) [29]. The KP cohort from Malaysia was also included [30].

The ILAS and LAST are ongoing cohort studies in Taiwan that target middle‐aged and older adults in Yuanshan Township (since 2011) and Taipei City (since 2016). These studies are aimed at investigating key aging‐related conditions, such as sarcopenia, frailty and cognitive function. The TLSA, initiated by the Health Promotion Administration (HPA), Ministry of Health and Welfare in Taiwan in 1989, is a nationally representative survey assessing physical, functional and mental health, along with demographic and household information through household interviews. Originally implemented for those aged 65 and above, it expanded to include those aged 50 and above in 1996 to better understand the aging process. The NAHSIT is a periodic survey in Taiwan led by Academia Sinica that focuses on the dietary habits, nutrition, health status and lifestyle of the Taiwanese population. It covers dietary intake, nutritional status, physical activity and other factors crucial for public health and nutrition. The Gan‐Dau Healthy Longevity Plan, initiated by Taipei Municipal Gan‐Dau Hospital, promotes healthy aging and community livability, aligning with the UN Decade of Healthy Aging [31, 32, 33].

The MUSCLE study is a cohort study of Japanese people aged 65 years and older in Maibara City since 2018 and includes detailed questionnaires of physical, oral and cognitive function. Tango study is a cohort study of middle‐aged and older Japanese individuals in Kyotango City and Ine Town since 2017, which includes questionnaires, medical checkups and physical fitness tests [28]. The NILS‐LSA is a longitudinal study of middle‐aged and older Japanese individuals from Obu City and Higashiura Town. It involves detailed questionnaires, medical checkups, anthropometric measurements, physical fitness tests and nutritional examinations. Follow‐ups occurred every 2 years until the seventh wave (July 2010 to July 2012), with new participants aged 40–79 years added yearly to replace those unable to attend follow‐up surveys. Additionally, data from a mixed cohort from Tsukuba University, primarily consisting of adolescents and young adults for certain muscle health metrics, are included in the analysis.

The KP cohort was carried out in the Kuala Pilah District, which is situated in Negeri Sembilan, one of Malaysia's 13 states. The district of Kuala Pilah was chosen for this study because it has the highest proportion of older adults among the seven districts in Negeri Sembilan. The demographics of Kuala Pilah's older population are similar to those of the Malaysian rural older population. Two waves of data collection with 12 months between them were undertaken among older adults aged 60 and above. The first wave commenced in 2013 and was completed in 2014, whereas the second wave was carried out from 2014 to 2015 [30].

All patient data are anonymized to protect confidentiality and identity.

### 2.2 Muscle Health Metrics

This retrospective cross‐sectional study utilized past data on muscle health metrics (Table S1), including calf circumference (centimetre), RASM (kilogram per square metre), handgrip strength (kilogram), five‐time chair stand (seconds) and gait speed (metre per second), based on the Asian Working Group for Sarcopenia (AWGS) 2019 recommendations [9]. Considering its applicability in certain populations, we also reported the ASM/BMI (kilogram/(kilogram per square metre)). Well‐trained study nurses or research associates conducted the measurements for all the parameters in all the cohorts of this study. The detailed measurement methods were described in Table S2.

The AWGS 2019 recommends measuring calf circumference (in centimetre) by wrapping a flexible, nonstretchable measuring tape around the widest point of the calf muscle, typically at the midcalf region. The measurement should be taken with the individual in a standing position and the feet flat on the ground. The RASM is calculated by dividing the ASM (muscle mass in the arms and legs), which is measured by bioelectrical impedance analysis (BIA) or dual‐energy x‐ray absorptiometry (DXA), by the square of an individual's height. The ASM/BMI (kilogram/(kilogram per square metre)) is calculated by dividing the ASM by the square of an individual's BMI. The screening tools of ASM used for each available cohort are listed in Table S3. For handgrip, the individual being tested should be in a seated position with their elbow flexed at 90° and their forearm in a neutral position. The individual then grips the dynamometer with maximum force, using their dominant hand, while keeping the hand and arm in a straight line. The measurement should be taken at least twice, and the highest reading is recorded. The cutoff values for handgrip strength to identify potential sarcopenia as per the AWGS 2019 guidelines are 28 kg for men and 18 kg for women. For the five‐time chair stand, the participants were asked to stand up from a chair with their arms folded across the chest five consecutive times as quickly as possible. The time taken to complete the task was measured via a handheld stopwatch and was used for the analyses. For gait speed, the individual is asked to walk at their usual pace, starting from a standing position at one end of the path and walking to the other end for a walking path of at least 4–6 m in length. The gait speed is then calculated by dividing the distance walked by the time taken to complete that distance in seconds. The formula is as follows: gait speed (metre per second) = distance (metres)/time (seconds).

### 2.3 Statistical Analyses

All descriptive analyses, including age‐ and sex‐specific normative, cutoff and mean and percentile values for muscle health metrics, were conducted. For calf circumference, RASM, ASM/BMI, handgrip strength and gait speed, the cutoff was defined as the 20th percentile; for the five‐time chair stand test, the cutoff was defined as the 80th percentile [2, 3].

Specifically, in this cross‐sectional study, participants were categorized into 5‐year age groups (e.g. 20–24, 25–29), allowing for comparison across age subgroups rather than tracking changes over time within individuals. Each age group represents an independent cross‐sectional snapshot of the population at different stages of life, rather than longitudinal measurements within the same individuals.

Joinpoint trend analysis software version 4.6.0.0 (National Cancer Institute, Bethesda, Maryland, United States) was used to examine trends in these cross‐sectional measurements. The starting age group for analysis varied by muscle health metric depending on data availability: Trends for calf circumference (centimetre) and handgrip strength (kilogram) were examined beginning in the 20s, for the five‐time chair stand (seconds) and gait speed (metre per second) beginning in the 30s and for RASM (kilogram per square metre) and ASM/BMI (kilogram/(kilogram per square metre)) beginning in the 40s. Annual percent changes (APCs) were estimated to represent the percent change per age group. The same regression models were employed to test differences between sex subgroups.

Joinpoint identifies time points when trends change significantly, using age (in 5‐year intervals) as the timescale. Joinpoint regression begins with a straight line (0 joinpoint) and tests whether adding more joinpoints is statistically significant, using the Monte Carlo Permutation method and the Bayesian Information Criterion (BIC) for model selection.

All analyses were conducted via SAS, version 9.4 (SAS Institute Inc., Cary, North Carolina, United States).

### 2.4 Role of the Funding Source

The study sponsors were not involved in designing the study; collecting, analysing or interpreting the data; writing the report; or the decision to submit the paper for publication.

## 3 Results

In total, data from 34 265 participants aged 20 years and older were collected for analysis. Demographics (age and gender), BMI and chronic conditions (not all cohorts had the same variables collected) are presented in Table S4. Cross‐sectional normative values for muscle health metrics in men and women, categorized by age group, are presented in Tables 1 and 2, respectively. The mean values, along with the 5th, 25th, 50th, 75th and 95th percentiles of calf circumference (centimetre), RASM (kilogram per square metre), handgrip strength (kilogram), five‐time chair stand (seconds) and gait speed (metre per second), are also delineated for both sexes in Table 3.

**TABLE 1:** Age‐specific normative values for metrics of muscle health among men.

Age group	Observations	Centiles					Mean (SD)
		5th	25th	50th	75th	95th	
Calf circumference (cm) (*n* = 6494)							
< 20	—	—	—	—	—	—	—
20–24	—	—	—	—	—	—	—
25–29	—	—	—	—	—	—	—
30–34	—	—	—	—	—	—	—
35–39	—	—	—	—	—	—	—
40–44	110	32.6	34.6	36.3	38.3	42.4	36.7 (3.1)
45–49	155	32.9	35.4	37.0	38.5	41.3	37.0 (2.5)
50–54	449	31.0	34.0	36.0	38.0	41.5	36.0 (3.1)
55–59	804	30.0	34.0	36.0	38.0	41.5	36.1 (3.2)
60–64	807	31.0	34.0	35.9	37.2	41.0	35.6 (3.1)
65–69	1294	30.0	33.5	35.5	37.5	40.6	35.5 (3.2)
70–74	1352	29.0	33.0	34.9	37.0	40.0	34.7 (3.4)
75–79	874	29.0	32.0	34.0	36.0	39.0	34.1 (3.3)
80+	649	28.0	31.0	33.1	35.5	38.9	33.3 (3.4)
Relative appendicular skeletal muscle by bioelectrical impedance analysis (kg/m^2^) (*n* = 1862)							
< 20	18	7.5	8.4	8.7	9.4	10.2	8.8 (0.7)
20–24	62	7.6	8.2	8.5	8.9	9.8	8.6 (0.8)
25–29	54	7.4	8.1	8.6	9.4	10.8	8.8 (1.1)
30–34	42	7.8	8.3	8.8	9.4	10.2	8.9 (0.8)
35–39	44	7.5	8.1	8.7	9.6	10.6	9.0 (1.1)
40–44	51	7.4	8.0	8.5	9.3	9.9	8.6 (0.9)
45–49	55	7.3	8.0	8.5	9.1	9.9	8.5 (0.8)
50–54	73	7.4	7.8	8.3	8.8	9.5	8.3 (0.7)
55–59	91	7.2	7.8	8.4	8.8	9.9	8.4 (0.9)
60–64	144	7.0	7.6	8.2	8.6	9.9	8.2 (0.9)
65–69	442	6.7	7.4	7.9	8.5	9.4	8.0 (0.8)
70–74	362	6.7	7.3	7.8	8.3	9.0	7.8 (0.8)
75–79	226	6.5	7.1	7.6	8.2	9.1	7.7 (0.8)
80+	198	5.9	6.9	7.4	7.9	9.0	7.4 (0.9)
Relative appendicular skeletal muscle by dual‐energy x‐ray absorptiometry (kg/m^2^) (*n* = 5956)							
< 20	15	5.6	6.6	7.3	8.3	10.0	7.4 (1.2)
20–24	160	6.4	7.1	7.8	8.5	10.2	7.9 (1.1)
25–29	196	6.2	7.1	7.8	8.7	10.0	8.0 (1.3)
30–34	167	6.2	7.4	8.0	8.7	10.2	8.1 (1.1)
35–39	167	6.8	7.4	8.2	9.0	10.0	8.2 (1.1)
40–44	293	6.4	7.2	7.9	8.6	10.0	8.0 (1.1)
45–49	342	6.4	7.2	7.8	8.5	9.6	7.9 (1.0)
50–54	570	6.4	7.3	7.9	8.5	9.5	7.9 (0.9)
55–59	747	6.5	7.3	7.9	8.4	9.3	7.9 (0.9)
60–64	777	6.4	7.2	7.7	8.2	9.1	7.7 (0.8)
65–69	942	6.2	7.0	7.6	8.2	9.0	7.6 (0.8)
70–74	694	6.1	6.8	7.3	7.9	8.8	7.4 (0.8)
75–79	485	5.9	6.6	7.1	7.7	8.5	7.1 (0.8)
80+	401	5.6	6.4	6.9	7.5	8.4	7.0 (0.9)
Handgrip strength (kg) (*n* = 7302)							
< 20	19	28.6	39.0	44.0	51.0	60.0	44.0 (9.3)
20–24	62	33.0	39.0	45.0	50.0	60.0	45.2 (8.3)
25–29	54	32.0	38.0	43.5	49.0	53.0	43.2 (6.7)
30–34	42	35.0	39.0	45.0	50.3	55.0	44.6 (6.9)
35–39	44	33.0	39.0	43.0	49.0	58.0	44.3 (7.7)
40–44	161	33.0	39.0	43.5	48.3	54.1	43.6 (6.4)
45–49	210	32.9	38.6	43.0	47.5	52.7	42.8 (6.3)
50–54	421	30.0	37.0	41.2	45.4	52.0	41.3 (6.5)
55–59	546	29.0	36.0	40.0	44.0	50.6	40.0 (6.4)
60–64	1005	22.7	32.0	37.0	41.4	47.8	36.3 (7.7)
65–69	1419	22.7	31.8	36.1	40.4	46.8	35.7 (7.2)
70–74	1414	22.0	30.0	34.3	38.4	44.5	33.9 (6.8)
75–79	1020	17.5	26.9	31.6	35.4	41.8	30.7 (7.4)
80+	885	14.5	24.3	28.7	32.4	38.0	27.8 (7.2)
Five‐time chair stand (s) (*n* = 3487)							
< 20	—	—	—	—	—	—	—
20–24	—	—	—	—	—	—	—
25–29	—	—	—	—	—	—	—
30–34	5	5.8	5.9	6.0	6.6	7.2	6.3 (0.6)
35–39	4	4.1	4.4	5.5	6.8	7.5	5.6 (1.5)
40–44	6	4.9	5.1	5.5	6.1	6.1	5.5 (0.5)
45–49	4	5.1	5.1	5.5	6.4	7.0	5.7 (0.9)
50–54	75	4.0	6.0	7.7	9.9	15.0	8.4 (3.3)
55–59	151	5.0	6.8	8.3	10.0	15.0	8.8 (2.8)
60–64	381	5.0	6.3	7.8	9.8	14.0	8.3 (3.1)
65–69	890	5.2	6.5	7.6	9.0	12.7	8.1 (2.4)
70–74	831	5.4	6.7	7.9	9.2	13.0	8.3 (2.4)
75–79	582	5.8	7.0	8.4	9.9	12.8	8.8 (2.7)
80+	558	6.3	7.9	9.3	11.5	15.0	10.0 (3.3)
Gait speed (m/s) (*n* = 6451)							
< 20	—	—	—	—	—	—	—
20–24	—	—	—	—	—	—	—
25–29	—	—	—	—	—	—	—
30–34	5	1.1	1.3	1.3	1.4	1.5	1.3 (0.2)
35–39	4	1.3	1.3	1.3	1.5	1.6	1.4 (0.2)
40–44	116	1.2	1.3	1.4	1.6	1.7	1.4 (0.2)
45–49	159	1.2	1.4	1.5	1.6	1.7	1.5 (0.2)
50–54	352	0.9	1.3	1.5	1.9	2.4	1.6 (0.5)
55–59	462	0.9	1.2	1.4	1.7	2.0	1.5 (0.5)
60–64	615	0.8	1.1	1.3	1.5	2.0	1.3 (0.4)
65–69	1534	0.7	1.0	1.2	1.4	1.7	1.2 (0.3)
70–74	1331	0.6	1.0	1.2	1.4	1.7	1.2 (0.3)
75–79	967	0.6	0.9	1.1	1.3	1.6	1.1 (0.3)
80+	906	0.5	0.8	1.0	1.2	1.5	1.0 (0.3)

**TABLE 2:** Age‐specific normative values for metrics of muscle health among women.

Age group	Observations	Centiles					Mean (SD)
		5th	25th	50th	75th	95th	
Calf circumference (cm) (*n* = 6715)							
< 20	—	—	—	—	—	—	—
20–24	—	—	—	—	—	—	—
25–29	—	—	—	—	—	—	—
30–34	—	—	—	—	—	—	—
35–39	—	—	—	—	—	—	—
40–44	103	30.3	32.4	34.0	35.9	40.0	34.3 (2.8)
45–49	165	31.0	32.6	34.0	35.6	39.0	34.3 (2.6)
50–54	523	29.0	32.0	34.0	36.0	39.0	34.0 (3.1)
55–59	877	29.0	32.0	34.0	36.0	39.8	34.1 (3.3)
60–64	892	29.0	32.0	33.5	35.5	39.0	33.7 (3.0)
65–69	1563	29.2	32.0	34.0	36.0	39.0	34.0 (3.1)
70–74	1233	28.0	31.0	33.0	35.0	38.6	33.2 (3.4)
75–79	809	27.0	30.3	32.5	34.5	38.0	32.5 (3.5)
80+	550	25.0	29.0	31.5	33.8	36.7	31.2 (3.7)
Relative appendicular skeletal muscle by bioelectrical impedance analysis (kg/m^2^) (*n* = 3010)							
< 20	10	6.5	6.7	6.8	7.3	8.7	7.1 (0.7)
20–24	39	6.3	6.6	7.0	7.4	8.2	7.0 (0.6)
25–29	32	6.2	6.6	6.9	7.5	8.4	7.0 (0.6)
30–34	48	6.2	6.5	6.9	7.4	7.9	7.0 (0.6)
35–39	38	5.8	6.4	6.6	6.7	7.7	6.6 (0.5)
40–44	72	5.8	6.2	6.7	6.9	7.8	6.7 (0.7)
45–49	109	5.7	6.2	6.4	6.8	7.6	6.5 (0.6)
50–54	158	5.5	5.9	6.3	6.7	7.2	6.3 (0.6)
55–59	218	5.4	5.8	6.1	6.5	7.4	6.2 (0.6)
60–64	338	5.2	5.7	6.1	6.5	7.1	6.1 (0.6)
65–69	804	5.2	5.7	6.0	6.4	7.1	6.1 (0.6)
70–74	586	5.1	5.6	6.0	6.4	7.1	6.1 (0.6)
75–79	347	5.1	5.7	6.1	6.5	7.1	6.1 (0.6)
80+	211	5.1	5.6	6.0	6.5	7.2	6.1 (0.7)
Relative appendicular skeletal muscle by dual‐energy x‐ray absorptiometry (kg/m^2^) (*n* = 6033)							
< 20	23	4.8	5.3	5.8	6.2	7.0	5.8 (0.7)
20–24	169	4.7	5.3	5.8	6.4	7.9	6.0 (1.0)
25–29	226	4.8	5.5	5.9	6.6	8.1	6.1 (1.0)
30–34	158	4.7	5.4	5.8	6.6	8.5	6.1 (1.1)
35–39	197	4.9	5.5	6.0	6.6	7.7	6.2 (0.9)
40–44	311	4.9	5.4	5.9	6.5	7.9	6.1 (0.9)
45–49	424	5.0	5.5	6.0	6.6	7.8	6.1 (0.9)
50–54	657	5.0	5.6	6.1	6.7	7.6	6.2 (0.8)
55–59	780	5.0	5.6	6.0	6.6	7.5	6.1 (0.8)
60–64	747	5.0	5.6	6.0	6.6	7.4	6.1 (0.8)
65–69	972	5.0	5.6	6.1	6.6	7.5	6.1 (0.8)
70–74	685	5.1	5.7	6.1	6.7	7.4	6.2 (0.7)
75–79	420	4.9	5.5	6.0	6.5	7.4	6.0 (0.8)
80+	264	4.8	5.6	5.9	6.3	7.1	5.9 (0.7)
Handgrip strength (kg) (*n* = 9428)							
< 20	13	19.1	22.0	24.2	28.0	35.0	25.1 (4.7)
20–24	39	19.0	24.0	26.0	31.0	37.0	26.9 (4.8)
25–29	32	19.0	24.5	27.0	29.5	35.0	27.0 (4.5)
30–34	48	20.0	22.0	26.0	30.0	36.0	26.2 (5.6)
35–39	38	18.0	22.0	26.5	29.0	35.0	25.7 (5.1)
40–44	175	19.0	23.4	26.1	29.0	33.3	26.5 (4.8)
45–49	272	20.5	23.8	26.0	28.7	34.0	26.3 (4.3)
50–54	560	17.0	21.9	24.8	28.0	32.9	24.9 (4.9)
55–59	737	17.0	21.2	24.0	27.0	32.0	24.2 (4.7)
60–64	1457	10.6	18.5	22.1	25.5	30.0	21.6 (5.8)
65–69	2087	11.9	19.0	22.2	25.2	29.5	21.8 (5.3)
70–74	1821	10.0	18.0	21.0	24.0	28.3	20.7 (5.6)
75–79	1301	7.6	16.0	19.9	23.0	27.0	19.0 (5.8)
80+	848	5.3	14.5	18.5	21.3	25.5	17.5 (6.3)
Five‐time chair stand (s) (*n* = 4298)							
< 20	—	—	—	—	—	—	—
20–24	—	—	—	—	—	—	—
25–29	—	—	—	—	—	—	—
30–34	8	4.0	4.6	5.9	6.4	7.3	5.6 (1.2)
35–39	4	5.5	5.7	6.4	6.9	6.9	6.3 (0.7)
40–44	9	4.4	5.3	5.4	5.5	6.3	5.4 (0.5)
45–49	11	5.5	5.6	5.9	6.1	10.4	6.2 (1.4)
50–54	103	5.1	6.7	8.0	9.6	14.0	8.4 (2.5)
55–59	193	5.0	7.0	8.9	10.0	13.3	8.7 (2.5)
60–64	514	5.0	6.4	7.7	9.6	13.0	8.2 (2.5)
65–69	1264	5.4	6.7	8.0	9.6	12.6	8.4 (2.7)
70–74	1021	5.4	6.9	8.1	9.7	13.5	8.6 (2.8)
75–79	690	5.6	7.0	8.4	10.0	14.0	9.0 (2.9)
80+	481	6.4	8.0	9.6	11.9	16.8	10.3 (3.3)
Gait speed (m/s) (*n* = 6970)							
< 20	—	—	—	—	—	—	—
20–24	—	—	—	—	—	—	—
25–29	—	—	—	—	—	—	—
30–34	8	1.1	1.3	1.3	1.5	1.6	1.4 (0.2)
35–39	4	1.4	1.4	1.4	1.4	1.4	1.4 (0.0)
40–44	112	1.1	1.2	1.3	1.4	1.6	1.3 (0.2)
45–49	176	1.2	1.3	1.4	1.5	1.7	1.4 (0.2)
50–54	408	0.9	1.2	1.5	1.7	2.0	1.5 (0.4)
55–59	532	0.9	1.2	1.4	1.5	2.0	1.4 (0.4)
60–64	697	0.8	1.2	1.3	1.5	2.0	1.3 (0.4)
65–69	1730	0.7	0.9	1.2	1.4	1.7	1.2 (0.3)
70–74	1483	0.6	0.9	1.2	1.4	1.6	1.2 (0.3)
75–79	1059	0.5	0.9	1.1	1.3	1.6	1.1 (0.3)
80+	761	0.4	0.7	1.0	1.2	1.4	0.9 (0.3)

**TABLE 3:** Age‐ and sex‐specific cutoff value for metrics of muscle health.

Age group	Men		Women	
	Observations	Cutoff value^a^	Observations	Cutoff value^a^
Calf circumference (cm)				
< 20	—	—	—	—
20–24	—	—	—	—
25–29	—	—	—	—
30–34	—	—	—	—
35–39	—	—	—	—
40–44	110	34.3	103	32.1
45–49	155	35.2	165	32.3
50–54	449	33.0	523	31.5
55–59	804	33.7	877	31.4
60–64	807	33.0	892	31.0
65–69	1294	33.0	1563	31.5
70–74	1352	32.0	1233	30.7
75–79	874	31.5	809	30.0
80+	649	30.5	550	28.0
Relative appendicular skeletal muscle by bioelectrical impedance analysis (kg/m^2^)				
< 20	18	8.3	10	6.7
20–24	62	8.2	39	6.5
25–29	54	8.0	32	6.4
30–34	42	8.2	48	6.5
35–39	44	8.0	38	6.1
40–44	51	8.0	72	6.1
45–49	55	7.9	109	6.0
50–54	73	7.7	158	5.8
55–59	91	7.6	218	5.8
60–64	144	7.5	338	5.6
65–69	442	7.3	804	5.6
70–74	362	7.2	586	5.5
75–79	226	7.0	347	5.6
80+	198	6.6	211	5.5
Relative appendicular skeletal muscle by dual‐energy x‐ray absorptiometry (kg/m^2^)				
< 20	15	6.5	23	5.2
20–24	160	6.9	169	5.2
25–29	196	6.9	226	5.4
30–34	167	7.2	158	5.2
35–39	167	7.3	197	5.4
40–44	293	7.1	311	5.3
45–49	342	7.1	424	5.4
50–54	570	7.2	657	5.5
55–59	747	7.2	780	5.5
60–64	777	7.0	747	5.5
65–69	942	6.9	972	5.5
70–74	694	6.7	685	5.5
75–79	485	6.4	420	5.4
80+	401	6.3	264	5.4
Handgrip strength (kg)				
< 20	19	34.0	13	21.2
20–24	62	38.0	39	23.0
25–29	54	36.0	32	24.0
30–34	42	38.0	48	22.0
35–39	44	38.0	38	21.5
40–44	161	38.0	175	23.0
45–49	210	37.0	272	23.0
50–54	421	36.0	560	21.0
55–59	546	35.0	737	20.8
60–64	1005	30.6	1457	17.3
65–69	1419	30.4	2087	18.1
70–74	1414	29.0	1821	17.0
75–79	1020	25.6	1301	15.0
80+	885	22.7	848	13.1
Five‐time chair stand (s)				
< 20	—		—	
20–24	—		—	
25–29	—		—	
30–34	5	6.9	8	6.5
35–39	4	7.5	4	6.9
40–44	6	6.1	9	5.8
45–49	4	7.0	11	6.1
50–54	75	10.5	103	10.0
55–59	151	10.0	193	10.5
60–64	381	10.0	514	10.0
65–69	890	9.4	1264	10.0
70–74	831	9.7	1021	10.2
75–79	582	10.3	690	10.5
80+	558	12.0	481	12.6
Gait speed (m/s)				
< 20	—		—	
20–24	—		—	
25–29	—		—	
30–34	5	1.2	8	1.2
35–39	4	1.3	4	1.4
40–44	116	1.3	112	1.2
45–49	159	1.3	176	1.3
50–54	352	1.2	408	1.2
55–59	462	1.1	532	1.2
60–64	615	1.0	697	1.0
65–69	1534	0.9	1730	0.9
70–74	1331	0.9	1483	0.9
75–79	967	0.9	1059	0.8
80+	906	0.8	761	0.6

^a^ For calf circumference, relative muscle index, hand grip, and gait speed, the cutoff was defined as the 20th percentile; for five‐time stand, the cutoff was defined as the 80th percentile.

### 3.1 Calf Circumference

**FIGURE 1:**
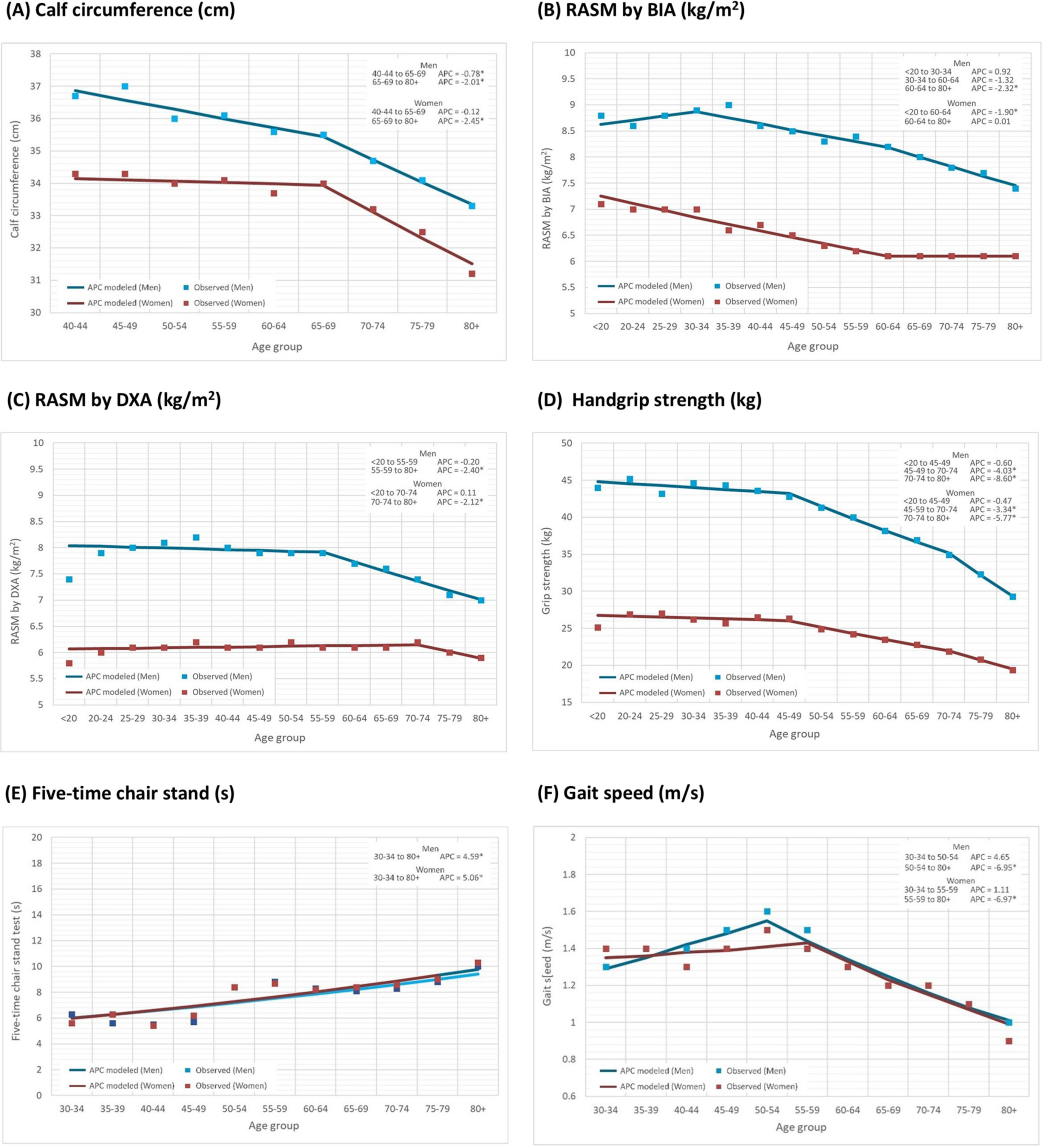
Mean values of age‐ and sex‐specific cross‐sectional normative metrics for muscle health. Annual percent change (APC), representing the percent change per age group here. *p < 0.05. RASM, relative appendicular skeletal muscle; BIA, bioelectrical impedance analysis; DXA, dual‐energy x‐ray absorptiometry.

**FIGURE 2:**
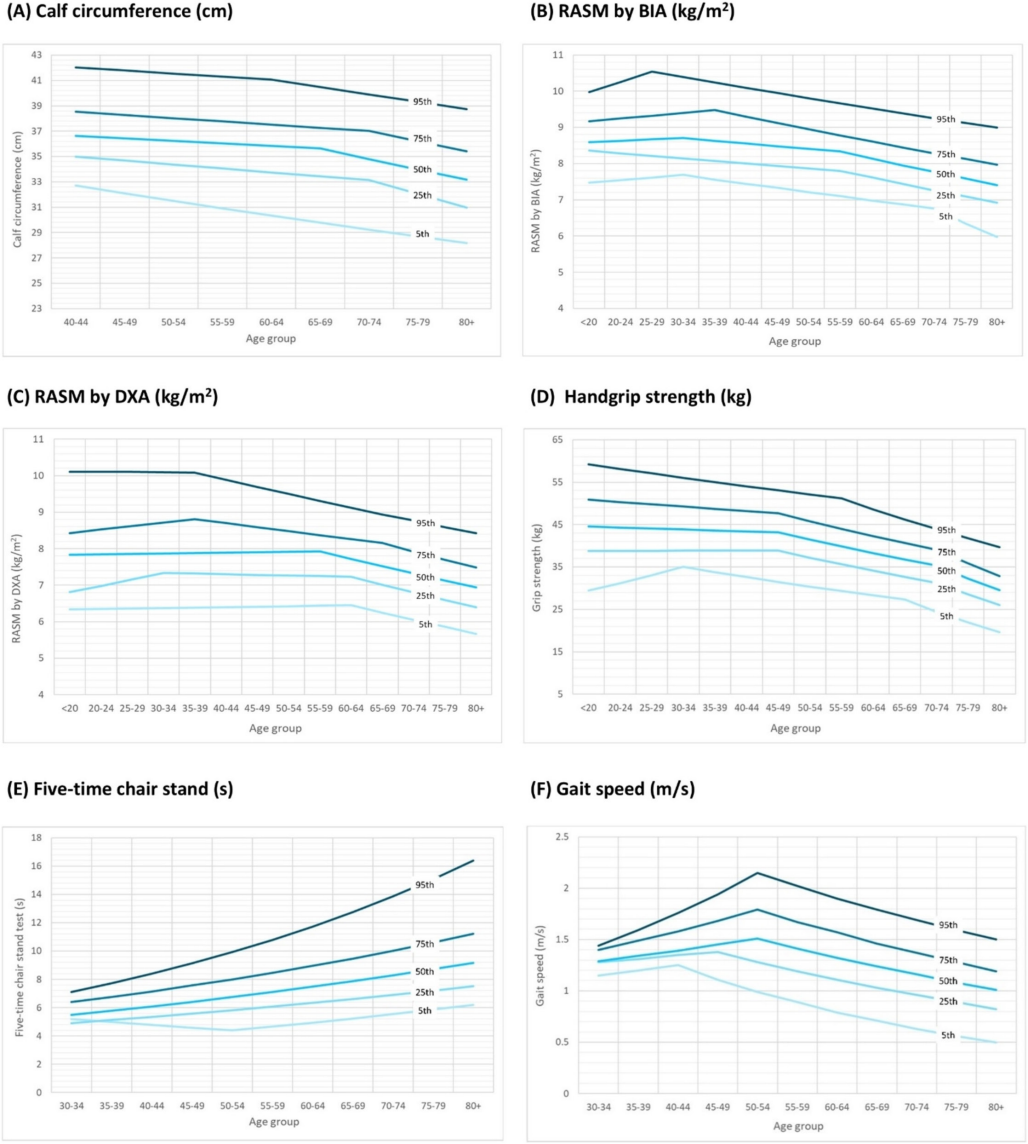
Age‐specific cross‐sectional percentiles for muscle health metrics among men. RASM, relative appendicular skeletal muscle; BIA, bioelectrical impedance analysis; DXA, dual‐energy x‐ray absorptiometry.

**FIGURE 3:**
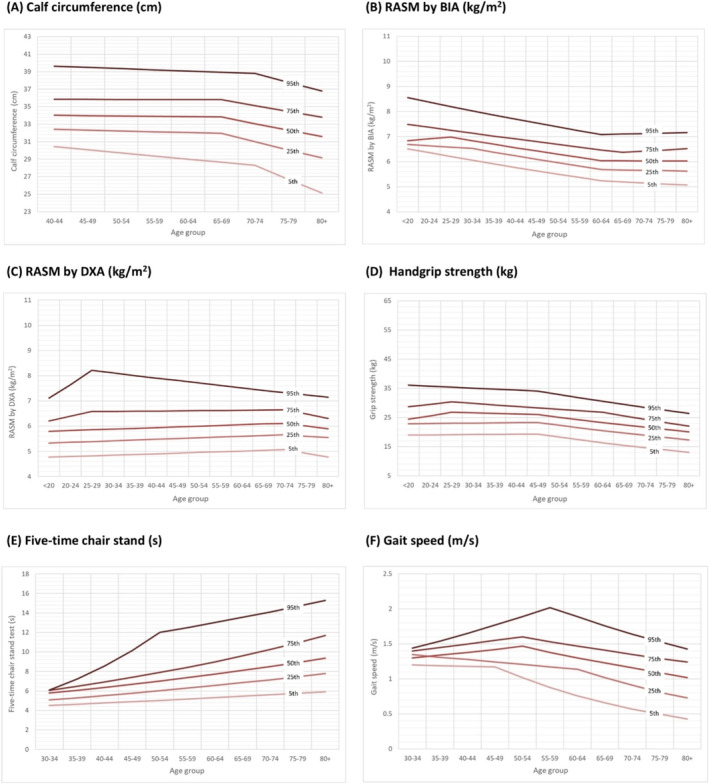
Age‐specific cross‐sectional percentiles for muscle health metrics among women. RASM, relative appendicular skeletal muscle; BIA, bioelectrical impedance analysis; DXA, dual‐energy x‐ray absorptiometry.

As a parameter associated with muscle mass, the mean [SD] value of calf circumference (centimetre) for men (*n* = 6494) in their 40s was 36.7 [3.1] cm for those aged 40–44 years and 37.0 [2.5] cm for those aged 45–49 years. These values were higher than those measured in women (*n* = 6715) of the same age (40–44 years: 34.3 [2.8] cm and 45–49 years old: 34.3 [2.6] cm). The mean calf circumference values remained relatively consistent up to the 65–69‐year age group, after which a decrease was noted in both sexes (Figure 1A). To facilitate the selection of appropriate cutoff points, the 20th percentile of calf circumference values was reported, with values of 33.0 cm for men and 31.5 cm for women in the 65–69‐year age group (Table 3). The age‐ and sex‐specific percentile calf circumference values are shown in Figures 2A and 3A, with more noticeable decreases observed in the lower percentile groups in both men and women.

### 3.2 RASM

The RASM, measured by either BIA or DXA, was higher in men than in women during adolescence. Cross‐sectional comparisons indicated that in men, peak RASM was observed in the 35–39‐year age group (Table 1), with lower values recorded in subsequent age groups. The inflection points were observed at ages 30–34 and 60–64 for BIA (Figure 1B) and at age 55–59 for DXA (Figure 1C). For women (Table 2), the pattern of RASM across age groups was less pronounced. Shifts in RASM values for women were observed in the 60–64 age group by BIA and 70–74 age group by DXA. The 20th percentiles of RASM values for men and women, as measured by either screening tool, are presented in Table 3 and Figures 2 and 3.

Combining the results from both screening tools (Table S5) shows that in men (*n* = 7818), a peak RASM of 8.4 [SD 1.1] kg/m^2^ was observed in the 35–39‐year age group. The RASM values in men remained relatively stable up to the age of 55–59 years (mean 8.0 [0.9] kg/m^2^), with lower values seen in older age groups (Figure S1). Conversely, the RASM in women remains relatively stable from their 20s onward (6.2 [1.0] kg/m^2^ vs. age 40–44:6.2 [0.9] kg/m^2^ and age 60–64:6.1 [0.7] kg/m^2^). The 20th percentiles of the RASM values were 7.0 kg/m^2^ for men and 5.5 kg/m^2^ for women aged 65–69 years (Table S6), which is compatible with the recommendations of the AWGS [9]. Age‐ and sex‐specific RASM percentiles (Figure S2) show that in men, the lower percentiles exhibited inflection points for RASM in younger age groups (e.g. 60–64 years for the fifth percentile) compared to the higher percentiles (e.g. 65–69 years for the 25th percentile), while no similar pattern was observed in women.

### 3.3 Body Mass Index‐Adjusted Appendicular Skeletal Muscle (ASM/BMI)

ASM/BMI was greater in men than in women (Table S7) across all age groups. In brief, ASM/BMI values remained relatively stable in both men and women from their 20s onward, with a decreased value observed in older age groups. Specifically, a marked decreased value in ASM/BMI was observed in women aged 35–39 years (Figure S3). The 20th percentile of ASM/BMI values was 0.78 kg/(kg/m^2^) for men and 0.56 kg/(kg/m^2^) for women aged 65–69 years (Table S8). Age‐ and sex‐specific percentile values for ASM/BMI revealed different inflection points of decline in the age‐ and sex‐specific percentile values (Figure S4).

### 3.4 Handgrip Strength

The values of handgrip strength in men (*n* = 7302, aged 20–24 years: 45.2 [8.3] kg) (Table 1) were significantly greater than those in women (*n* = 9428, aged 20–24 years: 26.9 [4.8] kg) (Table 2) in their 20s. A statistically significant decline in handgrip strength was observed in men aged 45–49 years (APC: −4.03) and 70–74 years (APC: −8.60). A similar trend was observed in women, with decreases noted at ages 45–49 years (APC: −3.34) and 70‐74 years (APC: ‐5.77) (Figure 1D). The 20th percentile of handgrip values was 30.4 kg for men and 18.1 kg for women aged 65–69 years (Table 3). Different inflection points of decline in handgrip strength were observed in the age‐ and sex‐specific percentile values (Figures 2D and 3D). An earlier inflection point for a decline in handgrip strength was found in men within the fifth percentile (30–34 years of age) than in those within the 95th percentile (55–59 years of age). For women, the inflection points of decline in handgrip strength were similar between those within the fifth percentile and those within the 95th percentile (at age 45–49).

### 3.5 Five‐Time Chair Stand Test

In terms of physical performance, the values of the five‐time chair stand test were remarkably similar in men (*n* = 3847) and women (*n* = 4298) throughout their lifespan (Figure 1E). An increased time taken to complete the test was observed in older age groups in both sexes. For men (Table 1), the mean value of the five‐time chair stand test was 6.3 [SD 0.6] seconds at the age of 30–34 years and then increased to 8.8 [2.7] seconds at the age of 75–79 years. For women (Table 2), the mean value of the five‐time chair stand test was 5.6 [1.2] seconds at the age of 30–34 years and then increased to 9.0 [2.9] seconds at the age of 75–79 years. The 20th percentiles of the five‐time chair stand test values were 9.4 s for men and 10.0 s for women aged 65–69 years (Table 3). Figures 2E and 3E show the age‐ and sex‐specific percentile values of the five‐time chair stand test.

### 3.6 Gait Speed

The peak gait speed observed in men (*n* = 6451) was 1.6 [1.5] m/s at the age of 50–54 years in this study (Table 1), with lower speeds recorded in subsequent age groups (Figure 1F; APC: −6.95). The gait speed in women (*n* = 6970) was slightly lower than that in men but remained constant from adolescence until the age of 55–59 years (Table 2; age 30–34 years: 1.4 [0.2] m/s vs. age 55–59 years: 1.4 [0.4] m/s) and declined thereafter (APC: −6.97) (Figure 1F). The 20th percentile of gait speed was 0.9 m/s for both men and women aged 65–69 (Table 3), which was similar to the AWGS consensus recommendations [9]. An earlier inflection point for a decline in gait speed was found in men within the fifth percentile (40–44 years of age) than in those within the 25th (at age 45–49), 50th (at age 50–54), 75th (at age 50–54) and 95th percentiles (at age 50–54). For women, the inflection points of decline in gait speed were also earlier in those within the fifth percentile (aged 45–49) than in those in the 95th percentile (aged 55–59) (Figures 2F and 3F).

## 4 Discussion

To the best of our knowledge, this is the first multinational study providing age‐ and sex‐specific cross‐sectional normative values of comprehensive muscle health metrics, including calf circumference (centimetre), RASM (kilogram per square metre), ASM/BMI (kilogram/(kilogram per square metre)), handgrip strength (kilogram), five‐time chair stand (seconds) and gait speed (metre per second). This study consolidated data from eight cohorts of 34 265 participants (16 164 men, 18 101 women) from Japan, Malaysia and Taiwan. The pooled analysis clearly outlined the age‐related changes in various muscle health metrics, with the inflection point of accelerated decline showing age‐ and sex‐specific characteristics. In terms of calf circumference, despite significant differences between men and women, the declining trends were similar, with the age of accelerated decline being approximately 65 years, corroborating previous reports [34, 35]. Conversely, RASM presented a unique pattern; men exhibited an accelerated decline at ages 55–59 years, whereas women remained relatively stable across decades. However, the ASM/BMI values were lower in older age groups for both sexes. Handgrip strength in both men and women demonstrated a similar declining trend, particularly after the age group of 45 years, despite differing baselines. A prior study that pooled data from 26 334 participants aged 65 years and older from eight cohorts proposed the use of 28 kg for men and 18 kg for women for the diagnosis of sarcopenia, utilizing the 20th percentile approach [36]. In terms of physical performance, the gait speed indicated that men exhibited a progressive increase and significantly decelerated after the age of 50, whereas women started this decline approximately 5 years later than men did. However, the rate of decline showed no sex differences. Furthermore, the time taken to perform a five‐time chair stand test displayed a consistent trend toward a progressive increase in both men and women. In the present study, disparities in muscle health metrics between genders were predominantly observed at baseline, a trend that may have been established since adolescence. However, the rates of age‐related decline in different muscle health metrics do not significantly differ, even at peak age, with the exception of usual gait speed and RASM. Interestingly, almost all muscle health metrics exhibited an analogous trend in which the top fifth percentile of participants demonstrated increased resistance to age‐related declines, whereas the lowest fifth percentile cohort experienced more rapid declines. These findings lend support to proactive preventative interventions aimed at establishing enhanced muscle mass and function at an earlier age.

In the majority of studies published in Western countries, both men and women have been reported to experience significant muscle loss after the age of 50 years [37, 38]. However, reports emanating from Taiwan, Japan, Korea and Hong Kong present a different trend, indicating that women do not experience a significant loss in muscle mass over time until they reach the age of 80 [39, 40] (S1, S2). Speculations surrounding this phenomenon are linked to the cohort effects or socioeconomic conditions prevalent in Asian countries in previous years (S1, S2). In recent decades, older women in Asia have generally maintained a relatively stable lifestyle, particularly in terms of housekeeping. A cross‐sectional study conducted in Korea revealed that older Korean women possess greater muscle mass than younger generations do (S2). Our study represents a cross‐sectional pooling of multiple cohorts; hence, we are unable to present data pertaining to longitudinal changes. Nevertheless, published data from Hong Kong and Japan spanning 4 years and 12 years, respectively, align with the findings of the current study (S1).

Intriguingly, calf circumference, as a potential surrogate marker of muscle mass, exhibited a consistent age‐related declining trend with an inflection point at the age group of 65 years, but the inflection points of calf circumference were not consistent with those of the RASM. Given the inability of DXA alone to differentiate intramuscular adiposity during the estimation of muscle mass, the constant muscle mass observed in women may be associated with an increase in intramuscular adiposity (S3, S4). Notably, Asian individuals are known to possess greater adiposity than Caucasians, a trend that persists even when older Asian individuals are underweight or malnourished [38]. Therefore, there is a pressing need for further research to develop more accurate measurements of muscle mass in older Asian women.

Gait speed presents another region‐specific finding, with Asian individuals seemingly walking faster than their Western counterparts. In contrast to the European Working Group for Sarcopenia in Older People, the AWGS recommends a higher cutoff to define slowness [9]. However, during the development of the AWGS diagnostic consensus, it was observed that the reported usual gait speed exhibited considerable variation across countries, which may be attributable to urban–rural differences. On the basis of this pooled analysis, gait speed progressively peaks at the age group of 50 and decelerates thereafter (with women commencing this deceleration approximately 5 years later than men). Notably, the time required to complete a five‐time chair stand test, another test for physical performance like 6‐m gait speed, progressively increases with age, with no sex differences.

Aging is an intricate process in which a multitude of factors coalesce, leading to adverse outcomes later in life, with skeletal muscle playing diverse roles throughout the lifespan, thereby engendering distinct impacts. While disability and dementia pose substantial risks, jeopardizing wellbeing later in life, various risk factors may contribute differently throughout the life course, with skeletal muscle serving as a prime exemplar. The upregulated microRNA miR‐29b‐3p, which is associated with muscle atrophy and is found in muscle cells, plasma and blood, is believed to mediate distal communication between muscle cells and neurons, influencing neuronal differentiation (S5). Exerkines are signalling molecules that are discharged in response to either acute or chronic exercise, and they exert their effects through endocrine, paracrine or autocrine pathways. These exerkines originate from various tissues, including skeletal muscle, heart, liver, adipose tissue and the nervous system, and they mediate numerous beneficial effects of exercise on cardiovascular, metabolic, immune and neurological health (S6). While all signalling molecules associated with physical activity, including exercise, are not solely restricted to skeletal muscle, it is important to note that all physical exercise is triggered by the action of skeletal muscle. Therefore, the maintenance of muscle health is highly important. To date, the optimal strategy for preserving muscle health during aging is the maintenance of an active lifestyle, coupled with the suitable adjustment of therapeutic agents utilized for various chronic conditions to prevent muscle wasting. Therefore, establishing a normative reference throughout one's lifespan is crucial for understanding the state of muscular health and implementing timely measures for healthy longevity. The recently published report from the Global Leadership Initiative for Sarcopenia underscores the importance of muscle strength, or muscle‐specific strength, being prioritized as the diagnostic element of sarcopenia (S7). In the present study, handgrip strength declined in both males and females during midlife and decreased over time, indicating that handgrip strength is a suitable indicator for promoting muscle health. Moreover, the decline in resistance of muscle health metrics exhibited by the top fifth percentile of individuals across the lifespan lends substantial support to the importance of enhancing muscle health at an earlier age and maintaining it over time.

A merit of our study is the inclusion of a large population ranging from young to very old individuals from Japan, Malaysia and Taiwan, particularly providing muscle health metrics cut‐off points for middle‐aged adults (Table S9). The joinpoint analysis also enabled us to identify the inflection points for each body composition variable as a function of age. Nevertheless, our study also has several limitations. First, the different recruitment algorithms used across the cohorts prevent us from linking these metrics to longitudinal outcomes. As a result, we used cross‐sectional normative values to assess age‐related changes in muscle health metrics. Future studies employing a longitudinal design will be necessary to capture follow‐up changes in these metrics. However, the muscle health metrics included in this study are highly standardized, making the pooled analysis reliable. Second, the number of participants in some age strata was relatively limited. As a result, the mean values for each muscle health metric in these groups may lack representativeness. However, our study still provides important insights into age‐ and sex‐specific cutoff points, and the broad age range of our participants allows us to reveal the trajectories of muscle health metrics throughout the aging process. Future research is warranted to increase the sample sizes of these age groups. Third, differences in urbanization levels between countries and regions may impact the results. Future studies should conduct more detailed analyses to explore differences between urban and rural areas. Finally, the present study lacks a suitable representative dataset for the development of normative values of the Short Physical Performance Battery, muscle composition (primarily intramuscular adiposity) and metabolic biomarkers (such as glucose or lipid profile) related to muscle health. Future studies should consider incorporating these data to provide a more comprehensive assessment of muscle health.

## 5 Conclusions

In this international multicohort study of 34 265 participants from Asia, we offer a comprehensive set of sex‐specific muscle health metrics with respect to age. All muscle health metrics exhibited declines with increasing age, though the inflection points of accelerated decline in different muscle health metrics varied; however, the top fifth percentile of individuals demonstrated increased resistance to declining trends, lending support to the importance of proactively promoting muscle health at an earlier age. We identify the critical junctures for each muscle health metric, which may aid in proactive preventative interventions and adjustments to the management of chronic conditions throughout the life course, thereby promoting healthy longevity for each individual.

## Disclosure

The funding source had no role in conducting this study, including the study design, data collection and analysis, manuscript preparation and review, or the decision to submit the manuscript for publication.

## Conflicts of Interest

The authors declare no conflicts of interest.

## Supporting information


**Table S1** The metrics of muscle health in each cohort.
**Table S2.** The test methods of each metric of muscle health.
**Table S3.** The screening tools of appendicular skeletal muscle mass.
**Table S4.** Participant characteristics in each cohort.
**Table S5.** Age‐ and sex‐specific normative values for relative appendicular skeletal muscle (RASM) measured by both bioelectrical impedance analysis (BIA) and dual‐energy x‐ray absorptiometry (DXA).
**Table S6.** Age‐ and sex‐specific cutoff value for relative appendicular skeletal muscle (RASM) measured by both bioelectrical impedance analysis (BIA) and dual‐energy x‐ray absorptiometry (DXA).
**Table S7.** Age‐ and sex‐specific normative values for BMI‐adjusted appendicular skeletal muscle mass (ASM/BMI) measured by both bioelectrical impedance analysis (BIA) and dual‐energy x‐ray absorptiometry (DXA).
**Table S8.** Age‐ and sex‐specific cutoff value for BMI‐adjusted appendicular skeletal muscle mass (ASM/BMI) measured by both bioelectrical impedance analysis (BIA) and dual‐energy x‐ray absorptiometry (DXA).
**Table S9.** Muscle metric cut‐off points for middle‐aged adults.
**Figure S1.** Mean cross‐sectional values of age‐ and sex‐specific relative appendicular skeletal muscle measured by both bioelectrical impedance analysis (BIA) and dual‐energy x‐ray absorptiometry (DXA).
**Figure S2.** Age‐specific cross‐sectional percentiles for relative appendicular skeletal muscle (RASM) measured by both bioelectrical impedance analysis (BIA) and dual‐energy x‐ray absorptiometry (DXA).
**Figure S3.** Mean cross‐sectional values of age‐ and sex‐specific BMI‐adjusted appendicular skeletal muscle mass (ASM/BMI) measured by both bioelectrical impedance analysis (BIA) and dual‐energy x‐ray absorptiometry (DXA).
**Figure S4.** Age‐specific cross‐sectional percentiles for BMI‐adjusted appendicular skeletal muscle mass (ASM/BMI) measured by both bioelectrical impedance analysis (BIA) and dual‐energy x‐ray absorptiometry (DXA).

## Data Availability

All potentially identifying data were encrypted to protect anonymity. Only investigators who receive approval from the principal investigator of each cohort and sign a data access agreement can obtain access to the data.

## References

[1] J. Campisi , P. Kapahi , G. J. Lithgow , S. Melov , J. C. Newman , and E. Verdin , “From Discoveries in Ageing Research to Therapeutics for Healthy Ageing,” Nature 571, no. 7764 (2019): 183–192. 31292558 10.1038/s41586-019-1365-2 PMC7205183

[2] C. López‐Otín , M. A. Blasco , L. Partridge , M. Serrano , and G. Kroemer , “Hallmarks of Aging: An Expanding Universe,” Cell 186, no. 2 (2023): 243–278. 36599349 10.1016/j.cell.2022.11.001

[3] L. K. Chen , “The Geriatrician's Call: Meeting the Needs of High‐Need, High‐Cost Older Individuals,” Aging Medicine and Healthcare 14, no. 2 (2023): 48–50.

[4] F. O. Asejeje and O. B. Ogunro , “Deciphering the Mechanisms, Biochemistry, Physiology, and Social Habits in the Process of Aging,” Archives of Gerontology and Geriatrics Plus 1 (2024): 100003.

[5] A. B. Newman , M. Visser , S. B. Kritchevsky , E. Simonsick , P. M. Cawthon , and T. B. Harris , “The Health, Aging, and Body Composition (Health ABC) Study‐Ground‐Breaking Science for 25 Years and Counting,” Journals of Gerontology. Series A, Biological Sciences and Medical Sciences 78, no. 11 (2023): 2024–2034. 37431156 10.1093/gerona/glad167 PMC10613019

[6] S. Kim and C. W. Won , “Sex‐Different Changes of Body Composition in Aging: A Systemic Review,” Archives of Gerontology and Geriatrics 102 (2022): 104711. 35588612 10.1016/j.archger.2022.104711

[7] L. K. Chen , “Connecting the Dots: Sarcopenia's Roles on Chronic Condition Management Toward Healthy Aging,” Journal of the Chinese Medical Association 87, no. 1 (2024): 3–4. 37991370 10.1097/JCMA.0000000000001027 PMC12718947

[8] L. K. Chen , “Editorial: Aging, Body Composition, and Cognitive Decline: Shared and Unique Characteristics,” Journal of Nutrition, Health & Aging 27, no. 11 (2023): 929–931.10.1007/s12603-023-2022-x37997711

[9] L. K. Chen , J. Woo , P. Assantachai , et al., “Asian Working Group for Sarcopenia: 2019 Consensus Update on Sarcopenia Diagnosis and Treatment,” Journal of the American Medical Directors Association 21, no. 3 (2020): 300–307.e2. 32033882 10.1016/j.jamda.2019.12.012

[10] L. K. Chen , H. Arai , P. Assantachai , et al., “Roles of Nutrition in Muscle Health of Community‐Dwelling Older Adults: Evidence‐Based Expert Consensus From Asian Working Group for Sarcopenia,” Journal of Cachexia, Sarcopenia and Muscle 13, no. 3 (2022): 1653–1672. 35307982 10.1002/jcsm.12981 PMC9178363

[11] H. Y. Lin , Y. C. Lin , L. K. Chen , and F. Y. Hsiao , “Untangling the Complex Interplay Between Social Isolation, Anorexia, Sarcopenia, and Mortality: Insights From a Longitudinal Study,” Journal of Nutrition, Health & Aging 27, no. 10 (2023): 797–805.10.1007/s12603-023-1993-y37960901

[12] S. S. Y. Yeung , E. M. Reijnierse , V. K. Pham , et al., “Sarcopenia and Its Association With Falls and Fractures in Older Adults: A Systematic Review and Meta‐Analysis,” Journal of Cachexia, Sarcopenia and Muscle 10, no. 3 (2019): 485–500. 30993881 10.1002/jcsm.12411 PMC6596401

[13] S. D. Anker , J. E. Morley , and S. von Haehling , “Welcome to the ICD‐10 Code for Sarcopenia,” Journal of Cachexia, Sarcopenia and Muscle 7, no. 5 (2016): 512–514. 27891296 10.1002/jcsm.12147 PMC5114626

[14] D. Rooks , T. Swan , B. Goswami , et al., “Bimagrumab vs Optimized Standard of Care for Treatment of Sarcopenia in Community‐Dwelling Older Adults: A Randomized Clinical Trial,” JAMA Network Open 3, no. 10 (2020): e2020836. 33074327 10.1001/jamanetworkopen.2020.20836 PMC7573681

[15] C. Becker , S. R. Lord , S. A. Studenski , et al., “Myostatin Antibody (LY2495655) in Older Weak Fallers: A Proof‐of‐Concept, Randomised, Phase 2 Trial,” Lancet Diabetes and Endocrinology 3, no. 12 (2015): 948–957. 26516121 10.1016/S2213-8587(15)00298-3

[16] Y. Shen , Q. Shi , K. Nong , et al., “Exercise for Sarcopenia in Older People: A Systematic Review and Network Meta‐Analysis,” Journal of Cachexia, Sarcopenia and Muscle 14, no. 3 (2023): 1199–1211. 37057640 10.1002/jcsm.13225 PMC10235889

[17] A. M. Negm , J. Lee , R. Hamidian , C. A. Jones , and R. G. Khadaroo , “Management of Sarcopenia: A Network Meta‐Analysis of Randomized Controlled Trials,” Journal of the American Medical Directors Association 23, no. 5 (2022): 707–714. 35183490 10.1016/j.jamda.2022.01.057

[18] B. K. Pedersen and M. A. Febbraio , “Muscles, Exercise and Obesity: Skeletal Muscle as a Secretory Organ,” Nature Reviews. Endocrinology 8, no. 8 (2012): 457–465. 10.1038/nrendo.2012.49 22473333

[19] C. Nelke , R. Dziewas , J. Minnerup , S. G. Meuth , and T. Ruck , “Skeletal Muscle as Potential Central Link Between Sarcopenia and Immune Senescence,” eBioMedicine 49 (2019): 381–388. 31662290 10.1016/j.ebiom.2019.10.034 PMC6945275

[20] C. M. Prado , F. Landi , S. T. H. Chew , et al., “Advances in Muscle Health and Nutrition: A Toolkit for Healthcare Professionals,” Clinical Nutrition 41, no. 10 (2022): 2244–2263. 36081299 10.1016/j.clnu.2022.07.041

[21] J. I. Yoo , H. Choi , and Y. C. Ha , “Mean Hand Grip Strength and Cut‐Off Value for Sarcopenia in Korean Adults Using KNHANES VI,” Journal of Korean Medical Science 32, no. 5 (2017): 868–872. 28378563 10.3346/jkms.2017.32.5.868 PMC5383622

[22] D. P. Leong , K. K. Teo , S. Rangarajan , et al., “Reference Ranges of Handgrip Strength From 125,462 Healthy Adults in 21 Countries: A Prospective Urban Rural Epidemiologic (PURE) Study,” Journal of Cachexia, Sarcopenia and Muscle 7, no. 5 (2016): 535–546. 27104109 10.1002/jcsm.12112 PMC4833755

[23] W. J. Lee , L. N. Peng , M. H. Lin , et al., “Six‐Year Transition of Physio‐Cognitive Decline Syndrome: Results From I‐Lan Longitudinal Aging Study,” Archives of Gerontology and Geriatrics 102 (2022): 104743. 35687948 10.1016/j.archger.2022.104743

[24] P. C. Yu , C. C. Hsu , W. J. Lee , et al., “Muscle‐to‐Fat Ratio Identifies Functional Impairments and Cardiometabolic Risk and Predicts Outcomes: Biomarkers of Sarcopenic Obesity,” Journal of Cachexia, Sarcopenia and Muscle 13, no. 1 (2022): 368–376. 34866342 10.1002/jcsm.12877 PMC8818605

[25] Y. C. Lin , H. Y. Lin , L. K. Chen , and F. Y. Hsiao , “Unveiling the Multifaceted Nexus of Subjective Aging, Biological Aging, and Chronological Age: Findings From a Nationally Representative Cohort Study,” Archives of Gerontology and Geriatrics 117 (2024): 105164. 37708578 10.1016/j.archger.2023.105164

[26] S. Y. Chuang , Y. L. Lo , S. Y. Wu , P. N. Wang , and W. H. Pan , “Dietary Patterns and Foods Associated With Cognitive Function in Taiwanese Older Adults: The Cross‐Sectional and Longitudinal Studies,” Journal of the American Medical Directors Association 20, no. 5 (2019): 544–550.e4. 30630727 10.1016/j.jamda.2018.10.017

[27] Z. J. Chen , F. P. Tang , S. Y. Chang , et al., “Resilience‐Happiness Nexus in Community‐Dwelling Middle‐Aged and Older Adults: Results From Gan‐Dau Healthy Longevity Plan,” Archives of Gerontology and Geriatrics 116 (2024): 105162. 37598465 10.1016/j.archger.2023.105162

[28] M. Yamada , Y. Kimura , D. Ishiyama , et al., “Combined Effect of Lower Muscle Quality and Quantity on Incident Falls and Fall‐Related Fractures in Community‐Dwelling Older Adults: A 3‐Year Follow‐Up Study,” Bone 162 (2022): 116474. 35752409 10.1016/j.bone.2022.116474

[29] S. T. Huang , C. Tange , R. Otsuka , et al., “Subtypes of Physical Frailty and Their Long‐Term Outcomes: A Longitudinal Cohort Study,” Journal of Cachexia, Sarcopenia and Muscle 11, no. 5 (2020): 1223–1231. 32558267 10.1002/jcsm.12577 PMC7567152

[30] W. Y. Choo , N. N. Hairi , R. Sooryanarayana , et al., “Elder Mistreatment in a Community Dwelling Population: the Malaysian Elder Mistreatment Project (MAESTRO) Cohort Study Protocol,” BMJ Open 6, no. 5 (2016): e011057. 10.1136/bmjopen-2016-011057 PMC4885447 27225651

[31] L. K. Chen , K. Iijima , H. Shimada , and H. Arai , “Community Re‐Designs for Healthy Longevity: Japan and Taiwan Examples,” Archives of Gerontology and Geriatrics 104 (2023): 104875. 36443116 10.1016/j.archger.2022.104875

[32] L. K. Chen , “Gan‐Dau Healthy Longevity Plan: The Community Model for Healthy Aging,” Aging Medicine and Healthcare 13, no. 3 (2022): 98–101.

[33] J. Amuthavalli Thiyagarajan , C. Mikton , R. H. Harwood , et al., “The UN Decade of Healthy Ageing: Strengthening Measurement for Monitoring Health and Wellbeing of Older People,” Age and Ageing 51, no. 7 (2022): afac147. 35776669 10.1093/ageing/afac147 PMC9249069

[34] F. Landi , R. Calvani , H. J. Coelho‐Junior , et al., “Estimated Appendicular Skeletal Muscle Mass Using Calf Circumference and Mortality: Results From the Aging and Longevity Study in the Sirente Geographic Area (ilSIRENTE Study),” Experimental Gerontology 169 (2022): 111958. 36150586 10.1016/j.exger.2022.111958

[35] S. E. Wu and W. L. Chen , “Calf Circumference Refines Sarcopenia in Correlating With Mortality Risk,” Age and Ageing 51, no. 2 (2022): afab239. 35134846 10.1093/ageing/afab239

[36] T. W. Auyeung , H. Arai , L. K. Chen , and J. Woo , “Letter to the Editor: Normative Data of Handgrip Strength in 26344 Older Adults ‐ A Pooled Dataset From Eight Cohorts in Asia,” Journal of Nutrition, Health & Aging 24, no. 1 (2020): 125–126.10.1007/s12603-019-1287-631886819

[37] B. H. Goodpaster , S. W. Park , T. B. Harris , et al., “The Loss of Skeletal Muscle Strength, Mass, and Quality in Older Adults: The Health, Aging and Body Composition Study,” Journals of Gerontology. Series A, Biological Sciences and Medical Sciences 61, no. 10 (2006): 1059–1064. 17077199 10.1093/gerona/61.10.1059

[38] D. Gallagher , S. B. Heymsfield , M. Heo , S. A. Jebb , P. R. Murgatroyd , and Y. Sakamoto , “Healthy Percentage Body Fat Ranges: An Approach for Developing Guidelines Based on Body Mass Index,” American Journal of Clinical Nutrition 72, no. 3 (2000): 694–701. 10966886 10.1093/ajcn/72.3.694

[39] H. Shimokata , F. Ando , A. Yuki , and R. Otsuka , “Age‐Related Changes in Skeletal Muscle Mass Among Community‐Dwelling Japanese: A 12‐Year Longitudinal Study,” Geriatrics & Gerontology International 14, no. Suppl 1 (2014): 85–92. 24450565 10.1111/ggi.12219

[40] L. K. Liu , W. J. Lee , C. L. Liu , et al., “Age‐Related Skeletal Muscle Mass Loss and Physical Performance in Taiwan: Implications to Diagnostic Strategy of Sarcopenia in Asia,” Geriatrics & Gerontology International 13, no. 4 (2013): 964–971. 23452090 10.1111/ggi.12040

