# Supplementary material for: Mapping Normative Muscle Health Metrics Across the Aging Continuum: A Multinational Study Pooling Data From Eight Cohorts in Japan, Malaysia and Taiwan
Source: J Cachexia Sarcopenia Muscle. 2025 Feb 19;16(1):e13731. doi: 10.1002/jcsm.13731 (PMC11839280; doi:10.1002/jcsm.13731)
Supplement: Supplementary file 1 — Table S1 The metrics of muscle health in each cohort. Table S2. The test methods of each metric of muscle health. Table S3. The screening tools of appendicular skeletal muscle mass. Table S4. Participant characteristics in each cohort. Table S5. Age‐ and sex‐specific normative values for relative appendicular skeletal muscle (RASM) measured by both bioelectrical impedance analysis (BIA) and dual‐energy x‐ray absorptiometry (DXA). Table S6. Age‐ and sex‐specific cutoff value for relative appendicular skeletal muscle (RASM) measured by both bioelectrical impedance analysis (BIA) and dual‐energy x‐ray absorptiometry (DXA). Table S7. Age‐ and sex‐specific normative values for BMI‐adjusted appendicular skeletal muscle mass (ASM/BMI) measured by both bioelectrical impedance analysis (BIA) and dual‐energy x‐ray absorptiometry (DXA). Table S8. Age‐ and sex‐specific cutoff value for BMI‐adjusted appendicular skeletal muscle mass (ASM/BMI) measured by both bioelectrical impedance analysis (BIA) and dual‐energy x‐ray absorptiometry (DXA). Table S9. Muscle metric cut‐off points for middle‐aged adults. Figure S1. Mean cross‐sectional values of age‐ and sex‐specific relative appendicular skeletal muscle measured by both bioelectrical impedance analysis (BIA) and dual‐energy x‐ray absorptiometry (DXA). Figure S2. Age‐specific cross‐sectional percentiles for relative appendicular skeletal muscle (RASM) measured by both bioelectrical impedance analysis (BIA) and dual‐energy x‐ray absorptiometry (DXA). Figure S3. Mean cross‐sectional values of age‐ and sex‐specific BMI‐adjusted appendicular skeletal muscle mass (ASM/BMI) measured by both bioelectrical impedance analysis (BIA) and dual‐energy x‐ray absorptiometry (DXA). Figure S4. Age‐specific cross‐sectional percentiles for BMI‐adjusted appendicular skeletal muscle mass (ASM/BMI) measured by both bioelectrical impedance analysis (BIA) and dual‐energy x‐ray absorptiometry (DXA). [file JCSM-16-e13731-s001.docx]

## Supplementary Table 1. The metrics of muscle health in each cohort

|  | Taiwan | | | | | Japan | | Malaysia |
| --- | --- | --- | --- | --- | --- | --- | --- | --- |
|  | ILAS | LAST | TLSA | NAHSIT | Gan-Dau | Mixed | NILS-LSA | KP |
| Cohort enrollment period | 2011  (Wave 1);  2018  (Wave 3) | 2016 | 1999 | 2013-2020 | 2022  (Wave 1) | 2018 | 2004-2006  (Wave 4) | 2013-2014  (Wave 1)  2014-2015  (Wave 2) |
| Calf circumference | ✓ | ✓ | ✓ | ✓ | - | - | ✓ | - |
| RASM or ASM/BMI | ✓ | ✓ | - | ✓ | - | ✓ | ✓ | - |
| Handgrip strength | ✓ | ✓ | - | ✓ | ✓ | ✓ | ✓ | ✓ |
| Five-time chair stand | ✓ | ✓ | - | - | - | ✓ | - | - |
| Gait speed | ✓ | - | - | ✓ | ✓ | ✓ | ✓ | - |

ILAS, I-Lan Longitudinal Aging Study; LAST, Longitudinal Aging Study of Taipei; TLSA, Taiwan Longitudinal Study of Aging; NAHSIT, Nutrition and Health Survey in Taiwan; Gan-Dau, Gan-Dau Healthy Longevity Plan; Mixed cohort, MUSCLE study and the Tango study; NILS-LSA, National Institute for Longevity Sciences, Longitudinal Study of Aging; KP, Kuala Pilah, Malaysia Cohort; RASM, relative appendicular skeletal muscle; ASM, appendicular skeletal muscle; BMI, body mass index

## Supplementary Table 2. The test methods of each metric of muscle health

1. Calf circumference

|  | Measurement method |
| --- | --- |
| Taiwan | |
| ILAS | Calf circumference was measured in centimeters using a measuring tape placed horizontally around the calf while the participant was seated, ensuring the tape was snug against the skin without compression. |
| LAST | Calf circumference was measured in centimeters using a measuring tape placed horizontally around the calf while the participant was seated, ensuring the tape was snug against the skin without compression. |
| TLSA | Calf circumference was measured in centimeters using a measuring tape placed horizontally around the calf while the participant was seated, ensuring the tape was snug against the skin without compression. |
| NAHSIT | Calf circumference was measured using a flexible measuring tape, primarily on the right side of the body, with measurements recorded accurately to the nearest 0.1 cm. |
| Japan | |
| NILS-LSA | Calf circumference was measured using a flexible measuring tape. Participants stood upright looking straight ahead with their arms relaxed at their sides. Measurements were taken where the measuring tape naturally touched the body without pulling it tight. In principle, measurements were taken on the right side. We took three measurements in total, discarded the first measurement, and used the average of the second and third measurements. Calf circumference was measured at the maximum girth of the calf, with measurements recorded to the nearest 0.1 cm. |

1. RASM or ASM/BMI

|  | Measurement method |
| --- | --- |
| Taiwan | |
| ILAS | A whole-body dual-energy X-ray absorptiometry (DXA) scan was carried out for each participant to measure body composition using a Lunar Prodigy instrument (GE Healthcare, Madison, WI, USA). ASM was defined by the sum of the lean soft tissue mass of four limbs, and the RASM was calculated as ASM divided by the squared body height (in meters). |
| LAST | Body composition was evaluated using bioimpedance analysis (BIA) (Inbody S10, Seoul, South Korea). ASM was obtained by summing the lean tissue mass of all four limbs, and the RASM was calculated as ASM divided by the squared body height (in meters). |
| NAHSIT | A professional radiologist operated the dual-energy X-ray absorptiometry (DXA) device (LUNAR PRODIGY, General Electric Company, Wisconsin, USA) to measure bone mineral density and perform body composition analysis of the whole body. ASM was derived from the examination.  Height and weight were measured using an automatic height and weight measuring device (Super-View HW-686, Kongho Instruments Co., Ltd, Taiwan). During the measurement, participants were instructed to wear the lightest possible clothing, remove shoes and socks, as well as any accessories and items from their pockets. |
| Japan | |
| Mixed | A bioelectrical impedance data acquisition system (MC-780A; TANITA Co, Ltd, Tokyo, Japan) was used to assess bioelectrical impedance. Participants stood on two metallic foot electrodes and gripped metallic hand electrodes. Using the segmental skeletal muscle mass, appendicular skeletal muscle mass (kg) was calculated. |
| NILS-LSA | Appendicular skeletal muscle (ASM) mass (kg), defined as the sum of lean tissue mass in the four limbs, was estimated using dual-energy x-ray absorptiometry (DXA; QDR-4500; Hologic, Bedford, MA, USA). Relative appendicular skeletal muscle (RASM) (kg/m^2^) was calculated as RASM = appendicular skeletal muscle mass (kg) / height^2^ (m^2^). |

1. Handgrip strength

|  | Measurement methods |
| --- | --- |
| Taiwan | |
| ILAS | Handgrip strength of the dominant hand was measured three times using dynamometer (“TOKYO” TTM ORIGINAL SMEDLAY’S DYNAMO METER 100 kg) while the participant was in a standing position and urged to use the greatest possible force. We used the highest value for the analysis in the current study. |
| LAST | Handgrip strength of the dominant hand was measured three times using a dynamometer (TTM Digital Hand Dynamometer) while the participant was in a seated position and urged to use the greatest possible force. We used the highest value for the analysis in the current study. |
| NAHSIT | Handgrip strength was measured using a dynamometer (JTECH, J-tech Medical, USA) while the participant was seated with feet shoulder-width apart and firmly on the ground. The elbow was relaxed, extended, and slightly positioned outward in an "A" shape, with the forearm and wrist maintained in a natural posture. The measurements were conducted alternately with the right and left hands, with each hand being tested three times. Participants were instructed to exert maximum effort for each trial, and a minimum of 30 seconds of rest was provided after each measurement for both hands. We used the highest value among the three trials with the dominant hand for the analysis in the current study. |
| Gan-Dau | Handgrip strength of the dominant hand was measured three times using dynamometer (TTM Digital Hand Dynamometer) while the participant was in a standing position and urged to use the greatest possible force. We used the highest value for the analysis in the current study. |
| Japan | |
| Mixed | Grip strength was assessed using a Smedley hand dynamometer, with the participant's arm positioned by their side. The participant squeezed the dynamometer with maximum effort, avoiding any additional body movement. The highest value from two trials for each hand was recorded as the representative measure. |
| NILS-LSA | Hand grip strength (kg) was the maximum grip strength over four trials for both hands (two trials for each hand) using a dynamometer (Takei T.K.K.5401 and T.K.K.4301a; Takei Scientiﬁc Instruments Co., Ltd, Tokyo, Japan). |
| Malaysia | |
| KP | Grip strength assessment was accomplished utilizing a dynamometer (JAMAR hand dynamometer 5030JI; Sammons Preston, Bolingbrook, IL, USA). Cut-offs for grip strength were based on the AWGS 2019 criteria: <28 kg in men and <18 kg in women. Prior to the measurement, the hand dynamometer was adjusted for different hand sizes. The participant was asked to stand with his/her shoulder adducted and neutrally rotated, elbow in full extension, forearm in a neutral position and wrist between 0 and 30° in dorsal flexion (handshake position). Measurement began with dominant hand followed by nondominant hand. Both hands were measured three times each, and the readings were recorded to the nearest 0·1 kg. The averages of the measurements of dominant hand were used in the analysis |

1. Five-time chair stand

|  | Measurement methods |
| --- | --- |
| Taiwan | |
| ILAS | The participant was seated in a stable, four-legged chair with a backrest placed against the wall. They were instructed not to lean against the backrest and to complete five sit-to-stand repetitions as quickly as possible. |
| LAST | The participant was seated in a stable four-legged chair with a backrest place against the wall. Participant was instructed not to lean against the backrest. Both hands were placed at the sides of the body, and the participant was instructed to complete a five-repetition sit-to-stand test as quickly as possible. |
| Japan | |
| Mixed | Participants were instructed to stand up and sit down five times as quickly as they could, with timing starting from the initial seated position and ending at the final standing position after the fifth repetition. |

1. Gait speed

|  | Measurement methods |
| --- | --- |
| Taiwan | |
| ILAS | Gait speed was measured by recording the time it took for the participant to walk a 6-meter distance (static start without deceleration) at their usual walking pace. The gait speed in this study was then calculated in meters per second (m/s). |
| NAHSIT | Gait speed was measured by recording the time it took for the participant to walk back and forth over a 4-meter distance at their usual walking speed. The gait speed in the current study was then calculated in meters per second (m/s). |
| Gan-Dau | Gait speed was measured by recording the time it took for the participant to walk a 6-meter distance (static start without deceleration) at their usual walking pace. The gait speed in this study was then calculated in meters per second (m/s). |
| Japan | |
| Mixed | Gait speed was assessed by timing how long it took each participant to walk a 5-meter distance at their normal walking pace. For this study, gait speed was then calculated in meters per second (m/s). |
| NILS-LSA | Gait parameters were evaluated on an 11-m straight walkway, including 1 m for acceleration and deceleration, at a usual speed. Gait speed (m/s) during normal gait was investigated using a walking analysis system (YW-3; Yagami Co., Aichi, Japan). Light sensors were placed at the start and end points to record the time to walk 10 m. Gait speed was measured by recording the time taken to walk 10 m, and this was evaluated through three trials. The final gait speed was calculated as the mean value of the three trials in meters per second (m/s). |

ILAS, I-Lan Longitudinal Aging Study; LAST, Longitudinal Aging Study of Taipei; TLSA, Taiwan Longitudinal Study of Aging; NAHSIT, Nutrition and Health Survey in Taiwan; Gan-Dau, Gan-Dau Healthy Longevity Plan; Mixed cohort, MUSCLE study and the Tango study; NILS-LSA, National Institute for Longevity Sciences, Longitudinal Study of Aging; KP, Kuala Pilah, Malaysia Cohort; RASM, relative appendicular skeletal muscle; ASM, appendicular skeletal muscle; BMI, body mass index

## Supplementary Table 3. The screening tools of appendicular skeletal muscle mass

|  | Taiwan | | | | | Japan | | Malaysia |
| --- | --- | --- | --- | --- | --- | --- | --- | --- |
|  | **ILAS** | **LAST** | TLSA | **NAHSIT** | Gan-Dau | **Mixed** | **NILS-LSA** | KP |
| BIA | - | ✓ | - | - | - | ✓ | - | - |
| DXA | ✓ | - | - | ✓ | - | - | ✓ | - |

ILAS, I-Lan Longitudinal Aging Study; LAST, Longitudinal Aging Study of Taipei; TLSA, Taiwan Longitudinal Study of Aging; NAHSIT, Nutrition and Health Survey in Taiwan; Gan-Dau, Gan-Dau Healthy Longevity Plan; Mixed cohort, MUSCLE study and the Tango study; NILS-LSA, National Institute for Longevity Sciences, Longitudinal Study of Aging; BIA, bioelectrical impedance analysis; DXA, dual-energy X-ray absorptiometry

## Supplementary Table 4. Participants characteristics in each cohort

|  | Taiwan | | | | | Japan | | Malaysia |
| --- | --- | --- | --- | --- | --- | --- | --- | --- |
|  | ILAS | LAST | TLSA | NAHSIT | Gan-Dau | Mixed | NILS-LSA | KP |
| Number | 2,840 | 1,544 | 4,440 | 12,761 | 841 | 7,205 | 2,221 | 2,413 |
| Age | | | | | | | | |
| <20 | - | - | - | 107 (0.8) | - | 32 (0.4) | - | - |
| 20-24 | - | - | - | 761 (6.0) | - | 102 (1.4) | - | - |
| 25-29 | - | - | - | 853 (6.7) | - | 86 (1.2) | - | - |
| 30-34 | - | - | - | 697 (5.5) | - | 90 (1.3) | - | - |
| 35-39 | - | - | - | 729 (5.7) | - | 82 (1.1) | - | - |
| 40-44 | - | - | - | 716 (5.6) | - | 124 (1.7) | 213 (9.6) | - |
| 45-49 | - | - | - | 825 (6.5) | - | 164 (2.3) | 320 (14.4) | - |
| 50-54 | 477 (16.8) | 56 (3.6) | 210 (4.7) | 920 (7.2) | 37 (4.4) | 176 (2.4) | 235 (10.6) | - |
| 55-59 | 599 (21.1) | 114 (7.4) | 700 (15.8) | 1,069 (8.4) | 90 (10.7) | 196 (2.7) | 285 (12.8) | - |
| 60-64 | 567 (20.0) | 210 (13.6) | 639 (14.4) | 1,075 (8.4) | 130 (15.5) | 671 (9.3) | 295 (13.3) | 647(26.8) |
| 65-69 | 494 (17.4) | 624 (40.4) | 555 (12.5) | 1,885 (14.8) | 179 (21.3) | 1,550 (21.5) | 246 (11.1) | 481 (19.9) |
| 70-74 | 384 (13.5) | 342 (22.2) | 1,061 (23.9) | 1,282 (10.1) | 192 (22.8) | 1,635 (22.7) | 259 (11.7) | 474 (19.6) |
| 75-79 | 206 (7.3) | 138 (8.9) | 731 (16.5) | 953 (7.5) | 114 (13.6) | 1,249 (17.3) | 201 (9.1) | 470 (19.5) |
| 80+ | 113 (4.0) | 60 (3.9) | 544 (12.3) | 889 (7.0) | 99 (11.8) | 1,048 (14.6) | 167 (7.5) | 341 (14.1) |
| Sex | | | | | | | | |
| Male | 1,393 (49.1) | 514 (33.3) | 2,358 (53.1) | 6,367 (49.9) | 287 (34.1) | 3,196 (44.4) | 1,141 (51.4) | 908 (37.6) |
| Female | 1,447 (50.9) | 1,030 (66.7) | 2,082 (46.9) | 6,394 (50.1) | 554 (65.9) | 4,009 (55.6) | 1,080 (48.6) | 1,505 (62.4) |
| BMI | 24.6±3.5 | 23.6±3.2 | 23.4±3.4 | 24.8±4.3 | 24.2±3.4 | 22.3±3.1 | 22.7±3.1 | 26.0±5.0 |
| Comorbidities* | | | | | | | | |
| Hypertension† | 1,010 (35.6) | 485 (31.5) | 1,478 (33.3) | - | 337 (40.1) | 351 (27.9) | 649 (29.2) | 1,271 (52.7) |
| Diabetes‡ | 387 (13.6) | 201 (13.0) | 638 (14.4) | - | 264 (31.4) | 94 (7.5) | 155 (7.0) | 643 (26.7) |
| Heart disease^§^ | 30 (1.06) | 105 (6.8) | 786 (17.7) | - | 219 (26.1) | 116 (9.2) | 272 (12.3) | 120 (5.0) |
| Pulmonary disease^#^ | 13 (0.5) | 63 (4.1) | 563 (12.7) | - | 149 (17.7) | 53 (4.2) | 195 (8.8) | 37 (1.5) |
| Kidney disease | 6 (0.2) | 59 (3.8) | 365 (8.2) | - | 112 (13.3) | 40 (3.2) | 65 (2.9) | - |

ILAS, I-Lan Longitudinal Aging Study; LAST, Longitudinal Aging Study of Taipei; TLSA, Taiwan Longitudinal Study of Aging; NAHSIT, Nutrition and Health Survey in Taiwan; Gan-Dau, Gan-Dau Healthy Longevity Plan; Mixed cohort, MUSCLE study and the Tango study; NILS-LSA, National Institute for Longevity Sciences, Longitudinal Study of Aging; BMI, body mass index

*For the Japan mixed cohort, data on comorbidities is available for only 1,260 individuals

†For Gan-Dau, all peripheral vascular system diseases are included.

‡For Gan-Dau, all endocrine and metabolic diseases are included.

^§^For ILAS, only coronary artery disease is included; for LAST, coronary artery disease, chronic heart failure, and cardiovascular disease are included; for NILS-LSA, coronary artery disease and chronic heart failure are included.

^#^For ILAS, only chronic obstructive pulmonary disease is included; for NILS-LSA, chronic obstructive pulmonary disease, asthma, pulmonary tuberculosis, and pleurisy are included.

## Supplementary Table 5. Age- and sex-specific normative values for relative appendicular skeletal muscle (RASM) measured by both bioelectrical impedance analysis (BIA) and dual-energy X-ray absorptiometry (DXA)

1. Among men

| **Age group** | **Observations** | Centiles | | | | | **Mean (SD)** |
| --- | --- | --- | --- | --- | --- | --- | --- |
|  |  | 5th | 25th | 50th | 75th | 95th |  |
| **RASM (kg/m^2^) (n=7,818)** | | | | | | | |
| <20 | 33 | 5.9 | 7.5 | 8.4 | 8.8 | 10.0 | 8.2 (1.2) |
| 20-24 | 222 | 6.5 | 7.3 | 8.1 | 8.7 | 9.9 | 8.1 (1.1) |
| 25-29 | 250 | 6.4 | 7.3 | 8.0 | 8.9 | 10.5 | 8.2 (1.3) |
| 30-34 | 209 | 6.4 | 7.5 | 8.2 | 9.0 | 10.2 | 8.2 (1.1) |
| 35-39 | 211 | 6.8 | 7.5 | 8.3 | 9.2 | 10.3 | 8.4 (1.1) |
| 40-44 | 344 | 6.5 | 7.4 | 8.0 | 8.7 | 10.0 | 8.1 (1.1) |
| 45-49 | 397 | 6.5 | 7.3 | 7.9 | 8.6 | 9.6 | 8.0 (1.0) |
| 50-54 | 643 | 6.4 | 7.4 | 8.0 | 8.5 | 9.5 | 8.0 (0.9) |
| 55-59 | 838 | 6.6 | 7.4 | 7.9 | 8.5 | 9.4 | 8.0 (0.9) |
| 60-64 | 921 | 6.5 | 7.2 | 7.7 | 8.3 | 9.1 | 7.8 (0.8) |
| 65-69 | 1,384 | 6.3 | 7.2 | 7.7 | 8.3 | 9.1 | 7.7 (0.8) |
| 70-74 | 1,056 | 6.2 | 7.0 | 7.5 | 8.1 | 8.9 | 7.5 (0.8) |
| 75-79 | 711 | 5.9 | 6.7 | 7.3 | 7.8 | 8.7 | 7.3 (0.9) |
| 80+ | 599 | 5.7 | 6.5 | 7.1 | 7.7 | 8.5 | 7.1 (0.9) |

1. Among women

| **Age group** | **Observations** | **Centiles** | | | | | **Mean (SD)** |
| --- | --- | --- | --- | --- | --- | --- | --- |
|  |  | **5th** | **25th** | **50th** | **75th** | **95th** |  |
| **RASM (kg/m^2^) (n=9,043)** | | | | | | | |
| <20 | 33 | 4.8 | 5.3 | 6.2 | 6.8 | 7.8 | 6.2 (0.9) |
| 20-24 | 208 | 4.9 | 5.4 | 5.9 | 6.8 | 8.1 | 6.2 (1.0) |
| 25-29 | 258 | 4.8 | 5.6 | 6.0 | 6.8 | 8.2 | 6.2 (1.0) |
| 30-34 | 206 | 4.9 | 5.5 | 6.1 | 6.9 | 8.4 | 6.3 (1.0) |
| 35-39 | 235 | 5.0 | 5.6 | 6.2 | 6.7 | 7.7 | 6.2 (0.9) |
| 40-44 | 383 | 5.0 | 5.6 | 6.1 | 6.7 | 7.9 | 6.2 (0.9) |
| 45-49 | 533 | 5.0 | 5.6 | 6.1 | 6.7 | 7.8 | 6.2 (0.8) |
| 50-54 | 815 | 5.1 | 5.7 | 6.2 | 6.7 | 7.6 | 6.2 (0.8) |
| 55-59 | 998 | 5.1 | 5.7 | 6.1 | 6.5 | 7.5 | 6.2 (0.7) |
| 60-64 | 1,085 | 5.0 | 5.6 | 6.0 | 6.5 | 7.4 | 6.1 (0.7) |
| 65-69 | 1,776 | 5.1 | 5.6 | 6.0 | 6.5 | 7.3 | 6.1 (0.7) |
| 70-74 | 1,271 | 5.1 | 5.6 | 6.1 | 6.6 | 7.3 | 6.1 (0.7) |
| 75-79 | 767 | 5.0 | 5.6 | 6.0 | 6.5 | 7.3 | 6.1 (0.7) |
| 80+ | 475 | 5.0 | 5.6 | 6.0 | 6.4 | 7.1 | 6.0 (0.7) |

## Supplementary Table 6. Age- and sex-specific cut-off value for relative appendicular skeletal muscle (RASM) measured by both bioelectrical impedance analysis (BIA) and dual-energy X-ray absorptiometry (DXA)

| **Age group** | **Men** | | **Women** | |
| --- | --- | --- | --- | --- |
|  | **Observations** | **20th percentile** | **Observations** | **20th percentile** |
| **RASM (kg/m^2^)** | | | | |
| <20 | 33 | 6.8 | 33 | 5.3 |
| 20-24 | 222 | 7.1 | 208 | 5.3 |
| 25-29 | 250 | 7.1 | 258 | 5.4 |
| 30-34 | 209 | 7.4 | 206 | 5.4 |
| 35-39 | 211 | 7.4 | 235 | 5.5 |
| 40-44 | 344 | 7.2 | 383 | 5.4 |
| 45-49 | 397 | 7.1 | 533 | 5.5 |
| 50-54 | 643 | 7.3 | 815 | 5.6 |
| 55-59 | 838 | 7.3 | 998 | 5.6 |
| 60-64 | 921 | 7.1 | 1,085 | 5.5 |
| 65-69 | 1,384 | 7.0 | 1,776 | 5.5 |
| 70-74 | 1,056 | 6.9 | 1,271 | 5.5 |
| 75-79 | 711 | 6.6 | 767 | 5.5 |
| 80+ | 599 | 6.4 | 475 | 5.5 |

## Supplementary Table 7. Age- and sex-specific normative values for BMI-adjusted appendicular skeletal muscle mass (ASM/BMI) measured by both bioelectrical impedance analysis (BIA) and dual-energy X-ray absorptiometry (DXA)

1. Among men

| **Age group** | **Observations** | Centiles | | | | | **Mean (SD)** |
| --- | --- | --- | --- | --- | --- | --- | --- |
|  |  | 5th | 25th | 50th | 75th | 95th |  |
| **ASM/BMI (kg/(kg/m^2^)) (n=7,818)** | | | | | | | |
| <20 | 33 | 0.77 | 1.01 | 1.12 | 1.20 | 1.35 | 1.09 (0.17) |
| 20-24 | 222 | 0.77 | 0.93 | 1.05 | 1.14 | 1.29 | 1.03 (0.16) |
| 25-29 | 250 | 0.80 | 0.91 | 1.01 | 1.11 | 1.25 | 1.02 (0.14) |
| 30-34 | 209 | 0.79 | 0.90 | 0.99 | 1.06 | 1.21 | 0.99 (0.12) |
| 35-39 | 211 | 0.77 | 0.90 | 0.97 | 1.05 | 1.20 | 0.98 (0.12) |
| 40-44 | 344 | 0.79 | 0.89 | 0.97 | 1.06 | 1.18 | 0.98 (0.12) |
| 45-49 | 397 | 0.80 | 0.88 | 0.95 | 1.03 | 1.14 | 0.96 (0.11) |
| 50-54 | 643 | 0.75 | 0.85 | 0.92 | 0.99 | 1.11 | 0.92 (0.11) |
| 55-59 | 838 | 0.71 | 0.83 | 0.90 | 0.97 | 1.10 | 0.90 (0.12) |
| 60-64 | 921 | 0.72 | 0.81 | 0.87 | 0.95 | 1.06 | 0.88 (0.11) |
| 65-69 | 1384 | 0.69 | 0.79 | 0.87 | 0.95 | 1.06 | 0.87 (0.11) |
| 70-74 | 1056 | 0.68 | 0.78 | 0.85 | 0.92 | 1.05 | 0.85 (0.11) |
| 75-79 | 711 | 0.66 | 0.75 | 0.82 | 0.89 | 1.01 | 0.83 (0.10) |
| 80+ | 599 | 0.64 | 0.73 | 0.80 | 0.87 | 0.99 | 0.80 (0.10) |

1. Among women

| **Age group** | **Observations** | **Centiles** | | | | | **Mean (SD)** |
| --- | --- | --- | --- | --- | --- | --- | --- |
|  |  | **5th** | **25th** | **50th** | **75th** | **95th** |  |
| **ASM/BMI (kg/(kg/m^2^)) (n=9,042)** | | | | | | | |
| <20 | 33 | 0.55 | 0.63 | 0.69 | 0.79 | 0.92 | 0.71 (0.11) |
| 20-24 | 208 | 0.56 | 0.64 | 0.70 | 0.79 | 0.90 | 0.72 (0.10) |
| 25-29 | 258 | 0.56 | 0.64 | 0.71 | 0.78 | 0.89 | 0.71 (0.10) |
| 30-34 | 206 | 0.56 | 0.64 | 0.69 | 0.78 | 0.89 | 0.71 (0.10) |
| 35-39 | 235 | 0.55 | 0.64 | 0.70 | 0.76 | 0.90 | 0.71 (0.10) |
| 40-44 | 383 | 0.56 | 0.63 | 0.69 | 0.76 | 0.86 | 0.70 (0.10) |
| 45-49 | 533 | 0.56 | 0.62 | 0.67 | 0.74 | 0.85 | 0.69 (0.09) |
| 50-54 | 815 | 0.52 | 0.59 | 0.65 | 0.72 | 0.84 | 0.66 (0.10) |
| 55-59 | 998 | 0.51 | 0.58 | 0.64 | 0.70 | 0.80 | 0.64 (0.09) |
| 60-64 | 1085 | 0.50 | 0.57 | 0.63 | 0.68 | 0.78 | 0.63 (0.09) |
| 65-69 | 1776 | 0.49 | 0.57 | 0.62 | 0.67 | 0.76 | 0.62 (0.08) |
| 70-74 | 1270 | 0.48 | 0.56 | 0.61 | 0.66 | 0.75 | 0.61 (0.08) |
| 75-79 | 767 | 0.47 | 0.54 | 0.59 | 0.65 | 0.72 | 0.59 (0.08) |
| 80+ | 475 | 0.46 | 0.52 | 0.57 | 0.62 | 0.72 | 0.57 (0.08) |

## Supplementary Table 8. Age- and sex-specific cut-off value for BMI-adjusted appendicular skeletal muscle mass (ASM/BMI) measured by both bioelectrical impedance analysis (BIA) and dual-energy X-ray absorptiometry (DXA)

| **Age group** | **Men** | | **Women** | |
| --- | --- | --- | --- | --- |
|  | **Observations** | **20th percentile** | **Observations** | **20th percentile** |
| **ASM/BMI (kg/(kg/m^2^))** | | | | |
| <20 | 33 | 0.96 | 33 | 0.61 |
| 20-24 | 222 | 0.89 | 208 | 0.62 |
| 25-29 | 250 | 0.90 | 258 | 0.62 |
| 30-34 | 209 | 0.88 | 206 | 0.62 |
| 35-39 | 211 | 0.88 | 235 | 0.63 |
| 40-44 | 344 | 0.87 | 383 | 0.62 |
| 45-49 | 397 | 0.86 | 533 | 0.61 |
| 50-54 | 643 | 0.83 | 815 | 0.58 |
| 55-59 | 838 | 0.81 | 998 | 0.57 |
| 60-64 | 921 | 0.79 | 1085 | 0.56 |
| 65-69 | 1384 | 0.78 | 1776 | 0.56 |
| 70-74 | 1056 | 0.76 | 1270 | 0.54 |
| 75-79 | 711 | 0.74 | 767 | 0.53 |
| 80+ | 599 | 0.72 | 475 | 0.51 |

## Supplementary Table 9. Muscle metric cut-off points for middle-aged adults

| **Age group** | **Men** | **Women** |
| --- | --- | --- |
|  | **Cut-off value*** | **Cut-off value*** |
| **Calf circumference (cm)** | | |
| 50-64 | <33.3 | <31.4 |
| **RASM by bioelectrical impedance analysis (kg/m^2^)** | | |
| 50-64 | <7.6 | <5.7 |
| **RASM by dual-energy X-ray absorptiometry (kg/m^2^)** | | |
| 50-64 | <7.2 | <5.5 |
| **ASM/BMI by bioelectrical impedance analysis (kg/kg/m^2^)** | | |
| 50-64 | <0.90 | <0.63 |
| **ASM/BMI by dual-energy X-ray absorptiometry (kg/kg/m^2^)** | | |
| 50-64 | <0.80 | <0.55 |
| **Handgrip strength (kg)** | | |
| 50-64 | <34 | <20 |
| **Five-time chair stand (s)** | | |
| 50-64 | ≥10.0 | ≥10.0 |
| **Gait speed (m/s)** | | |
| 50-64 | <1.2 | <1.2 |

*For calf circumference, RASM, ASM/BMI, hand grip, and gait speed, the cutoff was defined as the 20th percentile; for five-time stand, the cutoff was defined as the 80th percentile.

RASM, relative appendicular skeletal muscle; ASM, appendicular skeletal muscle; BMI, body mass index

## Supplementary Figure 1. Mean cross-sectional values of age- and sex-specific relative appendicular skeletal muscle measured by both bioelectrical impedance analysis (BIA) and dual-energy X-ray absorptiometry (DXA)


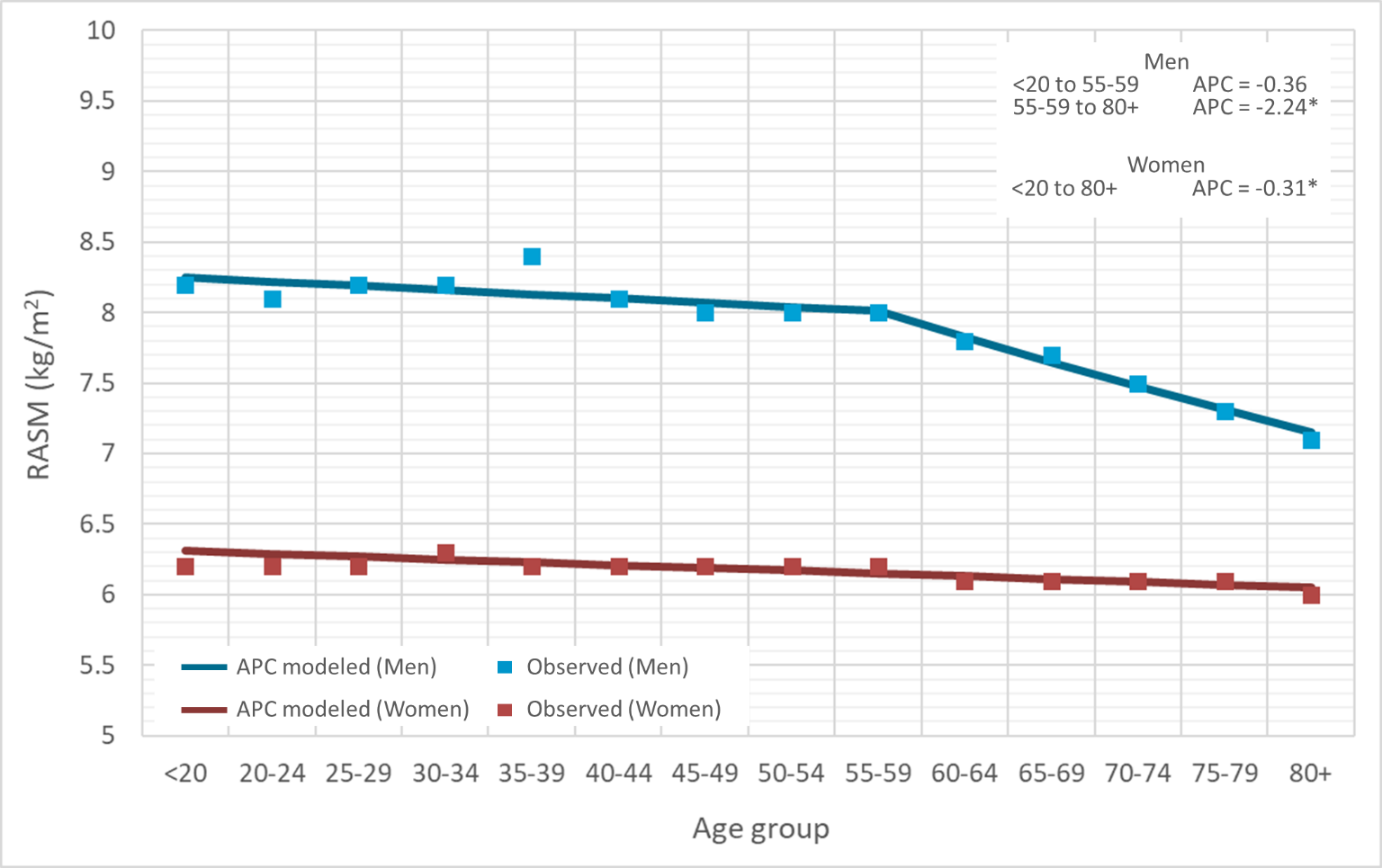


Annual percent change (APC), representing the percent change per age group here

*p<0.05

RASM, relative appendicular skeletal muscle; BIA, bioelectrical impedance analysis

## Supplementary Figure 2. Age-specific cross-sectional percentiles for relative appendicular skeletal muscle (RASM) measured by both bioelectrical impedance analysis (BIA) and dual-energy X-ray absorptiometry (DXA)

1. Among men


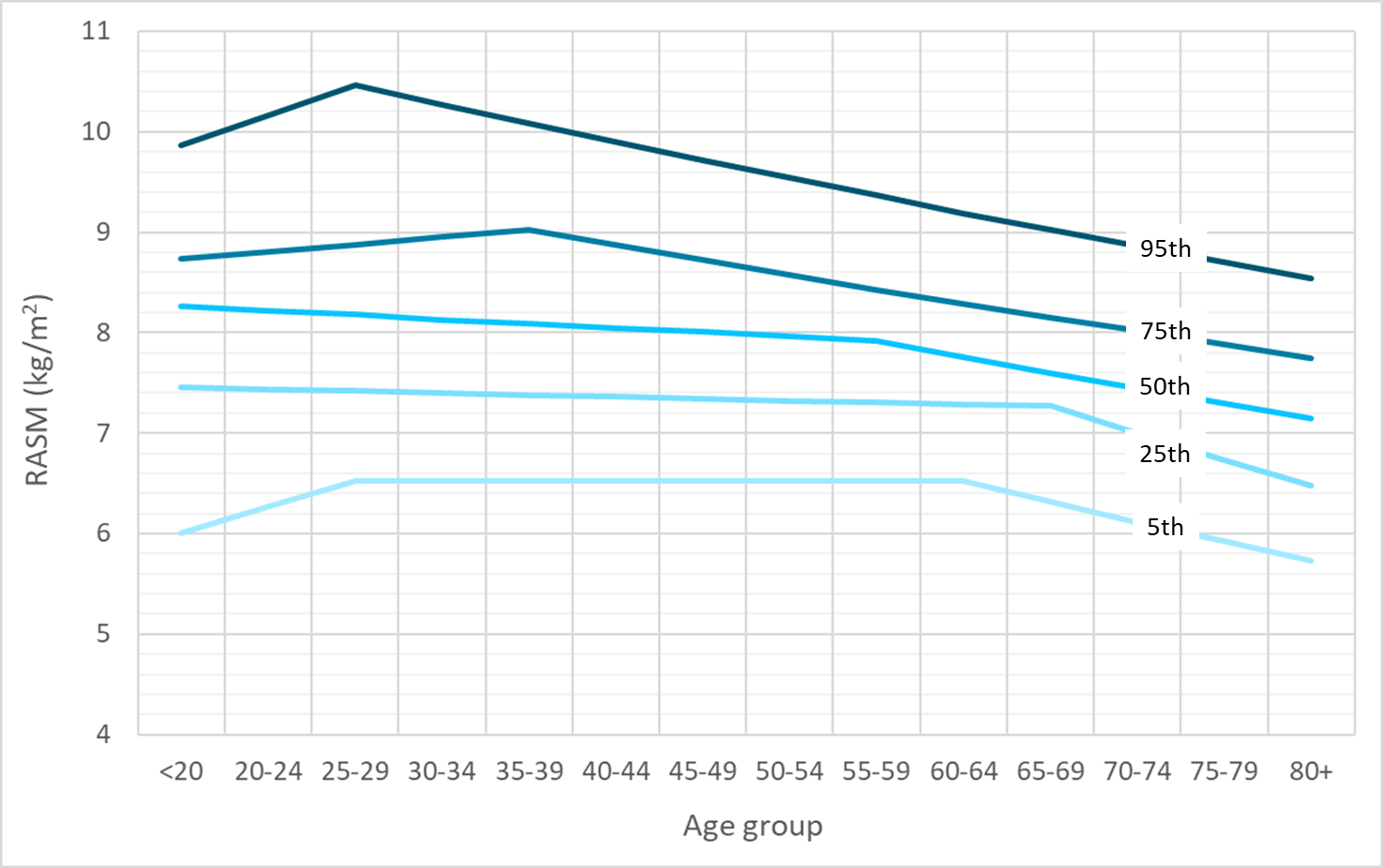


1. Among women


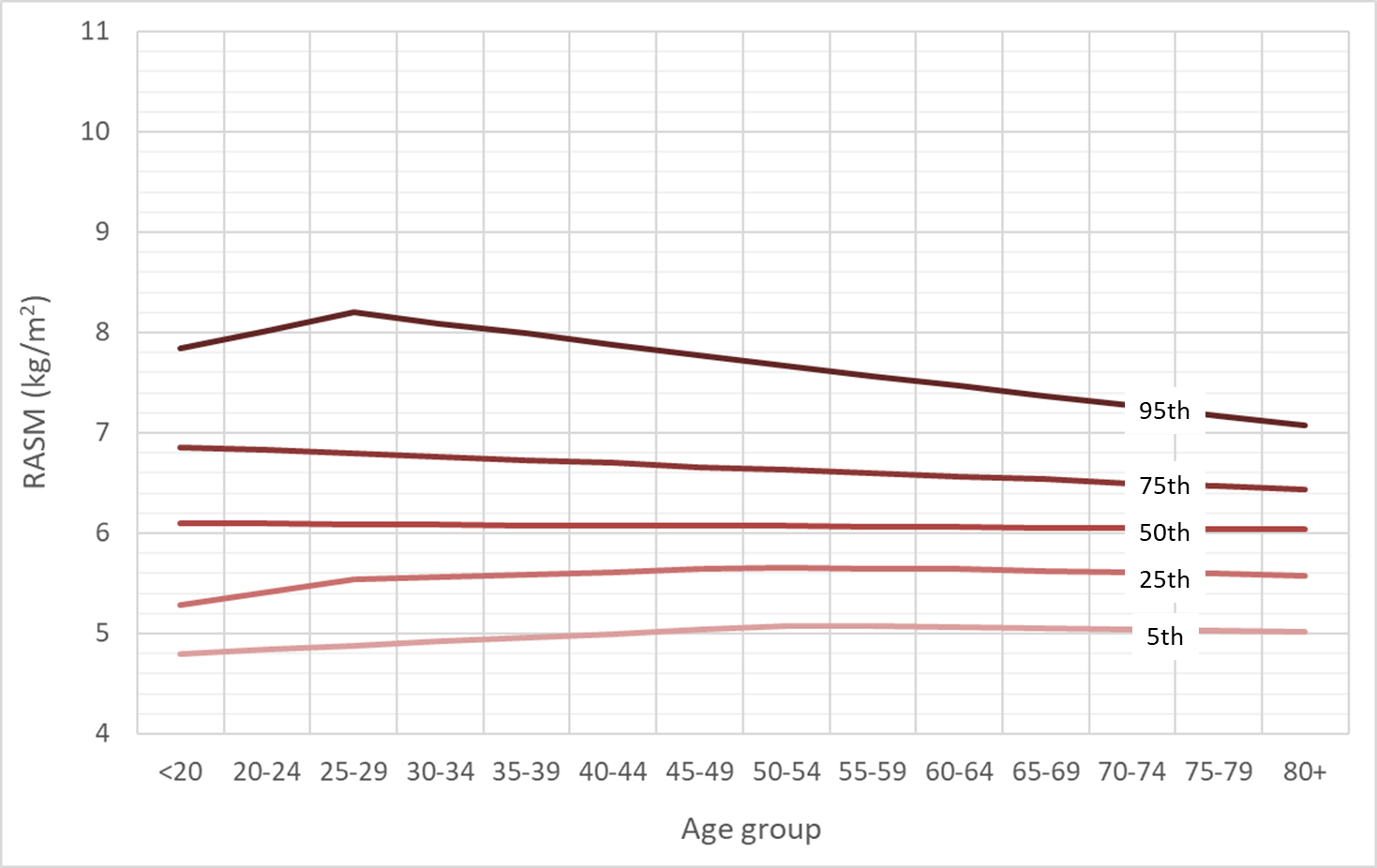


RASM, relative appendicular skeletal muscle; BIA, bioelectrical impedance analysis

## Supplementary Figure 3. Mean cross-sectional values of age- and sex-specific BMI-adjusted appendicular skeletal muscle mass (ASM/BMI) measured by both bioelectrical impedance analysis (BIA) and dual-energy X-ray absorptiometry (DXA)


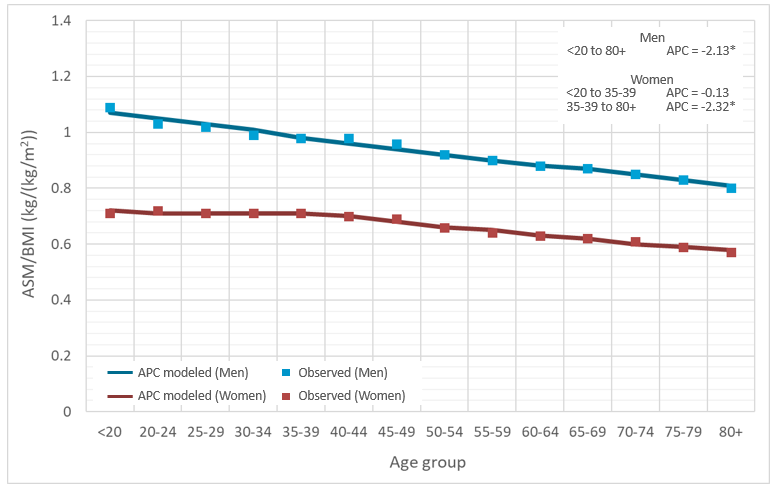


Annual percent change (APC), representing the percent change per age group here

*p<0.05

ASM, appendicular skeletal muscle; BMI, body mass index

## Supplementary Figure 4. Age-specific cross-sectional percentiles for BMI-adjusted appendicular skeletal muscle mass (ASM/BMI) measured by both bioelectrical impedance analysis (BIA) and dual-energy X-ray absorptiometry (DXA)

1. Among men


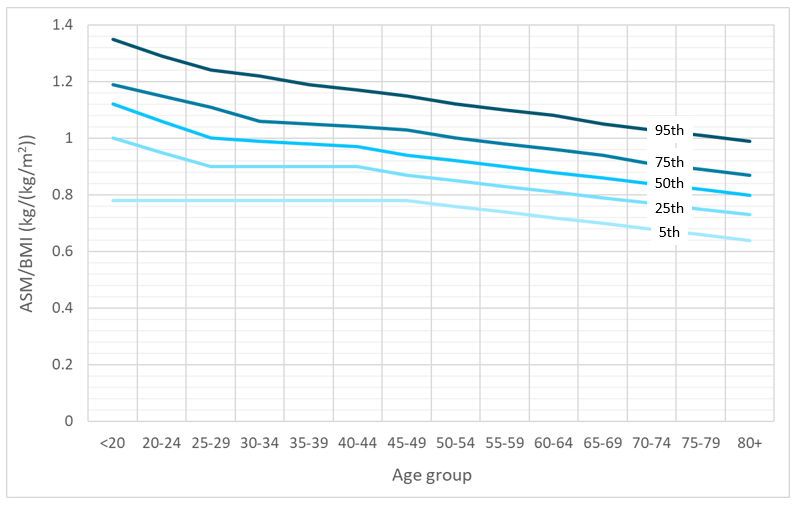


1. Among women


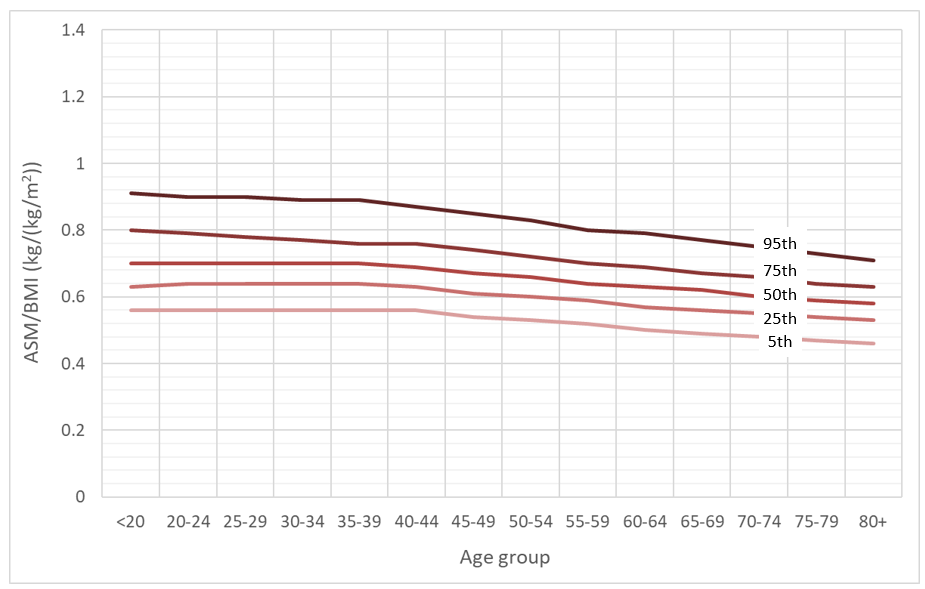


ASM, appendicular skeletal muscle; BMI, body mass index

## Supplementary References

S1. Auyeung TW, Lee SW, Leung J, Kwok T, Woo J. Age-associated decline of muscle mass, grip strength and gait speed: a 4-year longitudinal study of 3018 community-dwelling older Chinese. Geriatr Gerontol Int. 2014;14 Suppl 1:76-84.

S2. Hong S, Oh HJ, Choi H, et al. Characteristics of body fat, body fat percentage and other body composition for Koreans from KNHANES IV. J Korean Med Sci. 2011;26(12):1599-1605.

S3. Alavi DH, Henriksen HB, Lauritzen PM, et al. Quantification of adipose tissues by Dual-Energy X-Ray Absorptiometry and Computed Tomography in colorectal cancer patients. Clin Nutr ESPEN. 2021;43:360-368.

S4. Correa-de-Araujo R, Addison O, Miljkovic I, et al. Myosteatosis in the Context of Skeletal Muscle Function Deficit: An Interdisciplinary Workshop at the National Institute on Aging. Front Physiol. 2020;11:963. Published 2020 Aug 7.

S5. Yang CP, Yang WS, Wong YH, et al. Muscle atrophy-related myotube-derived exosomal microRNA in neuronal dysfunction: Targeting both coding and long noncoding RNAs. Aging Cell. 2020;19(5):e13107.

S6. Chow LS, Gerszten RE, Taylor JM, et al. Exerkines in health, resilience and disease. Nat Rev Endocrinol. 2022;18(5):273-289.

S7. Kirk B, Cawthon PM, Arai H, et al. The Conceptual Definition of Sarcopenia: Delphi Consensus from the Global Leadership Initiative in Sarcopenia (GLIS). Age Ageing. 2024;53(3):afae052.
